# Healthcare-related carbon footprinting—lower impact of a coronary stenting compared to a coronary surgery pathway

**DOI:** 10.3389/fpubh.2024.1386826

**Published:** 2024-08-21

**Authors:** Fabian Sack, Amanda Irwin, Raymond van der Zalm, Lorraine Ho, Danielle J. Celermajer, David S. Celermajer

**Affiliations:** ^1^Integrated Sustainability Analysis, School of Physics, The University of Sydney, Camperdown, NSW, Australia; ^2^Sydney Environment Institute, The University of Sydney Quadrangle, Camperdown, NSW, Australia; ^3^Performance Monitoring, Systems Improvement and Innovation Unit, Sydney Local Health District, Royal Prince Alfred Hospital, Stanmore, NSW, Australia; ^4^Faculty of Medicine and Health, Central Clinical School, Heart Research Institute, The University of Sydney, Newtown, NSW, Australia

**Keywords:** footprint, carbon emissions, greenhouse gases, patient care pathway, sustainability, stenting, bypass surgery, input–output analysis

## Abstract

Healthcare is a major generator of greenhouse gases, so consideration of this contribution to climate change needs to be quantified in ways that can inform models of care. Given the availability of activity-based financial data, environmentally-extended input–output (EEIO) analysis can be employed to calculate systemic carbon footprints for healthcare activities, allowing comparison of different patient care pathways. We thus quantified and compared the carbon footprint of two common care pathways for patients with stable coronary artery disease, with similar clinical outcomes: coronary stenting and coronary artery bypass surgery (CABG). Healthcare cost data for these two pathways were disaggregated and the carbon footprint associated with this expenditure was calculated by connecting the flow of money within the economy to the greenhouse gases emitted to support the full range of associated activities. The systemic carbon footprint associated with an average stable patient CABG pathway, at a large tertiary referral hospital in Sydney, Australia in 2021–22, was 11.5 tonnes CO_2_-e, 4.9 times greater than the 2.4 tonnes CO_2_-e footprint of an average comparable stenting pathway. These data suggest that a stenting pathway for stable coronary disease should be preferred on environmental grounds and introduces EEIO analysis as a practical tool to assist in health-care related carbon footprinting.

## 1 Introduction

Addressing the contribution of healthcare to climate change is a major current public health challenge. Global studies confirm that healthcare, a service industry with extensive supply chains, has a large carbon footprint and consequent climate change impact (1–3). This same high level of impact has also been demonstrated at the national level for many countries, including Australia (4). As a result, there is growing pressure on the healthcare sector to deliver reduction of emissions of greenhouse gases (GHG) such as carbon dioxide, methane, nitrous oxide, and a range of medical and industrial gases (5). In response to this pressure, work is now starting on factoring in GHG emissions in health-care decision making (6). For example, modelled on a UK example (7), the health department of New South Wales (Australia) is focused on improvements in energy and emissions efficiency at the facility level, addressing the state’s net zero emissions target (8). Similarly, as the Australian Government National Health and Climate Strategy (9) recognises, an understanding of the emissions generated across the full healthcare system to support a particular patient care pathway—its “carbon footprint”—could contribute to public health decision making by informing models of care optimised for both health and environmental benefits. While data alone are not sufficient to support systemic change, or changes at the level of medical practitioner decisions, readily accessible data on the emissions profiles of different procedures are a necessary condition for both. Particularly when combined with other policy and educational tools, it can support health system and clinical decision changes, to significantly lower emissions.

Global and national studies of the healthcare sector’s carbon footprint are usually derived from a modelling approach known as environmentally-extended input–output analysis (EEIO) (1–4). EEIO analysis quantifies the environmental impact associated with expenditure on goods and services using economic input–output tables. These tables contain information on the flow of money through the economy and the environmental impacts associated with the economic activity represented by that flow. By covering the whole economy, they are effectively “boundaryless.” As a result, EEIO can provide a comprehensive systemic footprint of any sector or activity accounted for in the input–output tables, including the healthcare sector. Recently there has been a study that extends the resolution of EEIO analysis to the level of an Australian state-based health sector organisation, using expenditure data sourced directly from internal accounts (10). This increase in resolution assists in bringing the carbon footprint into clinical decision-making and assists health managers to achieve sustainable value in healthcare (10). However, it does not provide insights into the carbon footprint of a particular patient care pathway.

There is a rapidly expanding literature looking to understand contributing activities to heath care carbon footprints. For the most part, analysis at this level employs methods that are variations on a different modelling approach to quantifying emissions, called unit process Life Cycle Assessment (LCA) (9, 11). LCA uses physical data on the relevant process collected from metering, observations, manufacturer’s specifications, and from detailed proprietary LCA databases, to assess the associated environmental impacts. By using physical data, LCA provides specific analysis on how emissions are generated, which can inform decision making in areas such as alternative product selection and optimising engineering specifications. This approach is being widely used in Australia, and has been used, for example, to estimate the carbon emissions of pathology testing (12), diagnostic imaging (6), and treatment of septic shock in an intensive care unit (13).

Because EEIO provides a sectoral analysis it is often characterised as a ‘top down’ carbon footprinting approach (9), contrasted with the ‘bottom up’ analysis of specific healthcare activities provided by LCA. This is usually taken to mean that EEIO is appropriate only for broad, jurisdictional and enterprise analysis and that LCA is the appropriate tool for detailed, activity-based analysis. However, a recent German study used EEIO derived emission factors to estimate the carbon footprints of hospital care pathways for an annual cohort of patients with acute decompensated heart failure at a German hospital (14). Building on this example, we propose that, given activity-based financial data, EEIO can also be employed to calculate systemic carbon footprints for quite specific healthcare activities, like surgical procedures. Moreover, we suggest that the use of EEIO at the activity level has certain advantages. Cumulatively these systemic footprints promise to account for the entire carbon footprint arrived at through national and enterprise carbon footprints. Unlike more tightly scoped LCA studies, these systemic footprints tend to be very much larger, incorporating the entire patient care pathway from admission to discharge, rather than a particular clinical procedure like surgery, or anaesthesia. These systemic footprints are also boundaryless, covering the entire extended supply chain that supports the healthcare activity in question. Irwin et al. estimate the extended supply chain (including postal, business and accounting services) accounts for 47% of Western Australian Health’s organisation’s carbon footprint, similarly Tennison et al. estimate the extended supply chain accounts for 62% of England’s National Health Service (NHS) footprint (3, 10). Further, as EEIO calculations are boundaryless and calculated using shared monetary units, they allow comparison of very different activities, whereas to arrive at comparative results, LCA studies need to be individually scoped to compare specific activities that share a functional unit. Finally, unlike LCA, which depends on sourcing physical data through resource intensive measurement, EEIO draws on readily available financial data and as such, can be more easily calculated. Given the urgency of measuring emissions across scales, this is an advantage, especially in cases where the emissions of different procedures are being compared. Our argument is not that EEIO should replace LCA, it is rather that EEIO analysis can usefully supplement LCA.

To exemplify how EEIO can be applied to calculate the systemic footprint of healthcare activities, and how these can be compared, this paper aims to quantify and compare the systemic carbon footprint of the patient care pathways associated with two common procedures with near-equivalent clinical outcomes. Our choice of example is informed by the fact that cardiovascular diseases in general, and coronary artery disease in particular, are the most common non-communicable diseases worldwide (11). However, as research into the climate impacts of cardiovascular healthcare is only just emerging (11), there is an absence of climate considerations in clinical guidance on the management of coronary artery disease, which do, however, indicate very important economic considerations. For example, the Australian Clinical Guidelines for the Management of Acute Coronary Syndromes do not mention climate considerations but do promote “cost-efficient improvement in patient outcomes as a result of new innovations in care” (15).

Many studies have compared clinical outcomes (survival, complications, quality of life) and economic costs of percutaneous transluminal coronary angioplasty (now nearly exclusively stenting); and coronary artery bypass graft surgery (CABG), in a wide range of disease states. In general, stenting is favoured in the setting of acute coronary syndromes and in stable single vessel disease, whereas surgery is more commonly favoured in diabetic patients and/or those with triple vessel disease (16–18). CABG and stenting generally share comparable clinical pathways prior to admission (diagnosis, referral and hospitalisation) and following discharge (ongoing monitoring) (19). They are also largely similar regarding survival outcomes and quality-of life measures (20). Thus, to ensure we chose an example with clinical equipoise, we compare the carbon footprints of stenting and CABG in stable, non-diabetic patients with two-vessel coronary disease (19). The argument we make in this paper is that the calculation of healthcare activity systemic carbon footprints can and should assist in introducing climate change considerations into decisions on the choice of patient care pathways.

## 2 Methods

### 2.1 Data

In using EEIO for their carbon footprint assessment of the National Health Service (NHS) in England, Tennison et al. note that a major limitation is that the “NHS’s internal spending information does not capture breakdown by economic sector and so was not used; instead, total spending on health care tracked by HM Treasury was proportioned using the transaction matrix in the UK MRIO model.”(3 p. 86–7). However, health care organisations in many jurisdictions globally now use activity based costing to help address economic efficiency and these data can be aligned to economic sectors (21). Australia uses activity-based funding in the healthcare sector whereby public hospitals get paid for the number and mix of patients they treat. To calculate the amount of the Australian Commonwealth Government’s payments to local hospital networks on an activity basis, the Independent Health and Aged Care Pricing Authority is responsible for determining the annual national efficient price and national efficient cost. To inform the calculation of these, Australian public hospitals routinely collect and report cost data as part of annual National Hospital Cost Data Collection (NHCDC) rounds (22). During each round, hospitals identify and classify all relevant costs into predetermined categories according to the NHCDC data request specifications and the Australian Hospital Costing Standards (23). These costs are then aggregated to cost buckets as specified in the relevant pricing framework (23). This process accounts for the direct and indirect costs of a care pathway, including specific procedures, from admission to discharge. This care pathway is referred to as a patient encounter. It includes all expenditure by the health care provider spent on this episode of care, including an apportionment for the general operation of the hospital and wider health system, and the specialised equipment required, such as a heart-lung machine or angiogram device. These cost bucket data can be aligned to economic sectors, enabling EEIO analysis.

Having restricted our sample to patient encounters associated with a pair of clinically equivalent procedures, we further minimised the variables in this novel application of the EEIO methodology by sampling a single hospital for 1 year to maximise the consistency of accounting practices that allocate expenditure to cost buckets. Cost data for NHCDC for the financial year 2021–22 (Round 26) were obtained from the Sydney Local Health District (SLHD) for costs incurred by Royal Prince Alfred Hospital (RPAH), a tertiary and quaternary referral hospital, for the elective surgical treatment of two-vessel coronary stenoses, by either:

1. Coronary artery bypass using 1 left internal mammary artery grafts (LIMA) graft: procedure code 38500–00; and coronary artery bypass using 1 saphenous vein graft: procedure code 38497–00 (*n* = 32)
2. Percutaneous insertion of > = 2 transluminal stents into multiple coronary arteries: procedure code 38306–02 (*n* = 29)

The SLHD had information by cost bucket by each procedure and by each patient, supplied for this analysis in the form of an average by cost bucket associated with each procedure. The cost data provided by SLHD were allocated to 16 different cost buckets for each procedure, according to the NHCDC Pricing Framework (23). Addressing the issue raised by Tennison et al. (3), the data were then disaggregated and allocated to sectors within Australia’s economy, informed by the Australian Hospital Patient Costing Standards (23), which provide detail on what expenditure types are accounted for in each cost bucket. Where costs from the same cost bucket were allocated to more than one economic sector, the allocation was weighted based on the ratio of expenditure on those sectors by the Australian healthcare sector in the 2021–22 financial year, which provided the industry average. The types and definitions of cost buckets are summarised in Table 1.

**Table 1:** Cost bucket definitions for data provided by SLHD and allocation of costs.

Cost bucket codes	Cost bucket description	% costs by cost bucket for CABG procedure	% costs by cost bucket for the stenting procedure
Allied	Allied Health: Average cost-includes clinical services which costs are recorded against the allied health cost centres.	2.12%	1.67%
Med	Medical: Average cost-includes the salaries and wages of medical officers including visiting medical officer (VMO) payments.	9.38%	4.83%
Nurse	Nursing: Average cost-includes the nursing salaries and wages of medical clinical service or ward cost centres.	7.04%	5.50%
Critical care	Critical care: Average cost-includes clinical services which costs are recorded against critical care cost centres as in adult intensive care unit (ICU), coronary care units, cardiothoracic ICU, high dependency units, neonatal & paediatric ICU, psychiatric ICU, special care nurseries.	20.97%	15.26%
Imag	Imaging: Average cost-includes services which costs are recorded against imaging cost centres and imaging costs recorded against an imaging account code.	1.87%	2.16%
OR	Operating Room/Theatre time (in minutes) is a combination of theatre duration and recovery duration.	20.89%	1.54%
Path	Pathology: Average cost-includes services which costs are recorded against pathology cost centres and pathology costs recorded against a pathology account code.	4.48%	3.13%
Pharm	Pharmacy: Average cost-includes services which costs are recorded against pharmacy cost centres and pharmacy costs recorded against a pharmacy account code.	0.66%	0.24%
Pros	Prosthetic: Average cost-includes services which costs are recorded against a prosthetic account code.	1.24%	9.30%
SPS	Specialist procedures suite: Average cost-includes all goods and services (excluding prosthesis), salaries and wages and VMO payments for SPS.	0.29%	32.92%
Ward & ED Supplies	Ward and emergency department (ED) supplies: Average cost-includes medical and surgical supplies and goods and services cost in clinical cost centres.	13.40%	2.69%
Non Clinical	Non-clinical: Average cost includes all costs associated with hotel (such as cleaning, linen and food), the salaries and wages of administrative and non-clinical staff.	5.94%	5.88%
On Cost	On cost: Average cost-includes superannuation and workers compensation costs.	4.91%	4.48%
Exclude	Includes costs which are not included in the pricing model and are excluded from the total cost in the portal such as Interest PPP, Depreciation and Actuarial Costs	3.54% + 1.26% Portal	2.13% + 1.27% Portal
Covid	COVID cost bucket includes the additional expense such as cleaning, security and PPE that has been due to the COVID-19 pandemic	1.47%	1.63%
PatTrans	Patient Transport cost bucket. Average cost-includes the costs associated with patient transportation.	0.51%	5.36%
Total %		100%	100%

These cost data required disaggregation to the 1,284 economic sectors provided in the single-region input–output table built using the Australian Industrial Ecology Laboratory (23). This disaggregation was executed by building a concordance matrix, allocating the total expenditure through the matrix, and excluding costs that are not included in final demand within the input–output table, which would otherwise result in double counting. These steps are detailed below.

### 2.2 Step 1—building a concordance matrix

**Figure 1:**
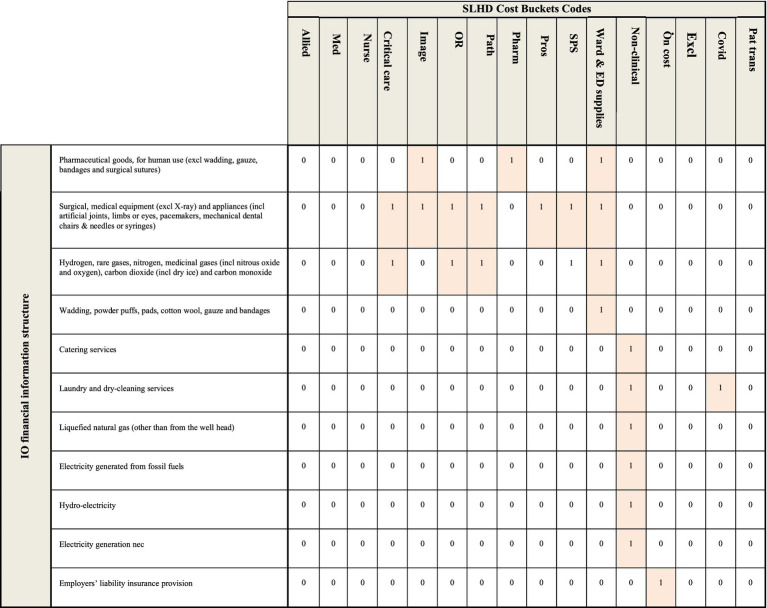
An extract of the concordance used to connect the expenditure information provided by the SLHD and the financial information contained within the input–output table. A value of 1 represents a connection between the sector in the row and the sector in the column, a value of 0 designates no connection between the sector in the row and the sector in the column.

A binary concordance matrix was built based on the details included in NSW Health’s accounting framework. A concordance is a binary matrix which connects two different sets of information, in this case the sector structure used to report economy-wide transactions in the input–output table, and the cost bucket structure used to report the SLHD cost data. An extract of the concordance used for this study is included in Figure 1, where a value of 1 in a cell represents a connection between the data reported within the input–output table (in the rows) and the cost bucket data received from SLHD (in the columns).

Assumptions made in the creation of this concordance include:

- Any maintenance and repair costs are matched to the non-clinical cost bucket;
- The pathology, pharmacy, critical care and imaging cost buckets includes the cost of relevant staff;
- Higher value capital purchases such as medical equipment and furniture are matched to the cost bucket where they will be used, such as OR and SPS;
- Non-medical equipment and furniture are matched to the non-clinical cost bucket;
- Consumable items are matched to the Ward & ED supplies cost bucket if they are used for the clinical care of patients (e.g., bandages); and
- Consumable items are matched to the non-clinical cost bucket if they are not directly used in the clinical care of patients (e.g., cleaning supplies).

### 2.3 Step 2—allocating the total expenditure

Where expenditure in one cost bucket was connected to more than one input–output sector, for example the non-clinical cost bucket, the percentage used to allocate the total expenditure in that cost bucket to the relevant input–output sectors was determined using the Australian healthcare industry average expenditure across each of the component sectors. The data for this was sourced from the intermediate demand matrix within the 2021–2022 input–output table. This meant, for example, that 1.6% of the costs recorded under the non-clinical cost bucket were allocated to expenditure against the electricity generation sectors. At this point, all costs included in each cost bucket were allocated to one or more sectors within the input–output table.

### 2.4 Step 3—excluding costs not covered in final demand

A final adjustment was made to the expenditure values allocated to each of the economic sectors within the input–output table to exclude any expenditure that did not constitute final demand within them. This includes expenditure on salaries and wages, which is captured in the value-added matrix in the input–output table. The exclusion of any expenditure that did not constitute final demand extends also to capital expenditure, present in the cost bucket data as depreciation, an accounting mechanism for allocating capital expenditure that has already been incurred. This expenditure was assigned by SLHD to the ‘Excluded’ bucket. This exclusion resulted in an underestimate of the systemic footprint, since it did not include emissions associated with expenditure on capital items during the 2021–22 financial year. This is recognised as a limitation of our method, which might be best described as an operating systemic carbon footprint. Both these adjustments are routinely made in studies of this sort (14).

### 2.5 Analysis—environmentally-extended input–output analysis

Environmentally-extended input–output (EEIO) analysis, a well-established methodology for calculating carbon footprints (4), was used to calculate the carbon footprint for these two cardiac procedures. This methodology, introduced by Wassily Leontief (24), relies on matrix calculations to connect the flow of money within a given economy, represented in an input–output table, to the greenhouse gases emitted by activities in that economy. This approach has been widely used to understand carbon footprints across the global economy in recent decades but is still novel as a tool for measuring patient care pathways (1, 4). Key to these calculations is Equation 1, which connects the economic transactions captured in the input–output table to the environmental impact generated by those economic transactions.


(1) footprint=qLy

Here, **q** represents the direct intensities vector, providing information on the greenhouse gases emitted for every dollar of total output for each sector in the input–output table. The **L** matrix, also known as the Leontief inverse, provides information on the economic interdependencies within and between all sectors within the economy by quantifying the value of total input required from each sector to produce one dollar worth of output for a given sector. It is derived using Equation 2, where **I** represents the identity matrix (the matrix equivalent of the number 1) and **A** represents the direct requirements matrix. The direct requirements matrix **A** is calculated from the data within the input–output table by dividing each transaction in the intermediate demand matrix, which provides information on the value of transactions between sectors within the economy, by the total output for each sector.


(2) $$L={\left(I-A\right)}^{-1}$$

Finally, the **y** vector in Equation 1 provides information on the expenditure data associated with the entity for which the footprint is being calculated. In this case, two **y** vectors were created, one each for the CABG and stenting procedures. These expenditure vectors were built by adjusting the disaggregated cost bucket data to exclude depreciation and direct salaries and wages. These transactions are accounted for elsewhere in the input–output tables from which **q** and **L** are derived, and their inclusion in the expenditure vector used to calculate the footprint for each procedure would inflate the results.

A single-region input–output table for the 2021–2022 financial year was built using the Australian Industrial Ecology Laboratory (25). This input–output table contained information on the economic transactions within and between 1,284 sectors across the Australian economy, and the associated greenhouse gas emissions generated by each sector during the year, drawn from Australia’s National Greenhouse Inventory Report in line with emissions estimation rules adopted under the Paris Agreement (26). The full list of sectors in the Industrial Ecology Laboratory is given in the Supplementary material.

All greenhouse gases were included in this analysis, but adjustments associated with land use change were excluded.

## 3 Results—systemic carbon footprint of two cardiac procedures

For the 2021–22 financial year, 29 patients with stable two-vessel coronary disease underwent stenting, with an average total cost of $16,820 per patient, while 32 patients with the same diagnosis underwent the CABG procedure, with an average total cost of $73,211 per patient, 4.4 times that of the stenting procedure. Once expenditure on items not accounted for as a component of final demand within the input–output table was excluded, the average cost for those patients who underwent CABG was $38,196, 3.4 times more than that of the $11,150 cost for those who had a stenting procedure. Clinical decision making was according to physician and patient preferences. There were some differences of note in the internal cost allocation to cost buckets for each procedure, with, for example, the Specialist Procedures Suite cost bucket receiving the highest allocation for stenting (33%), and the Critical Care cost bucket receiving the highest allocation for CABG (21%). The two right-hand columns in Table 1 provide the percentage allocation of costs between cost buckets for each procedure.

**Figure 2:**
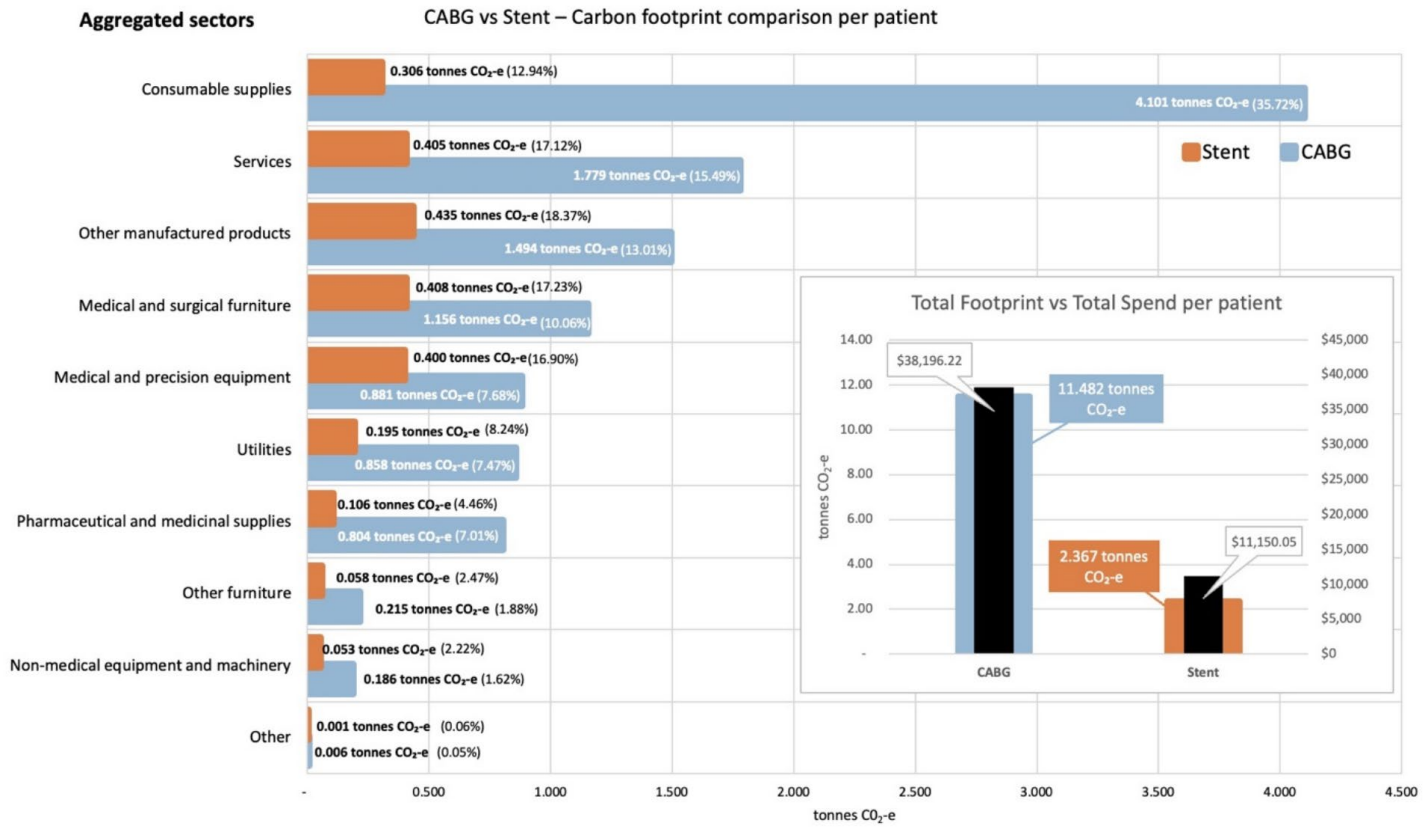
Carbon footprint contribution of each aggregated sector by weight and percentage for activities associated with CABG and stenting procedures, per average patient. Inset shows total carbon footprint and total spend for activities associated with each procedure, per average patient.

From these data, we calculated that the carbon footprint associated with the average CABG procedure was 11.5 tonnes CO_2_-e, 4.9 times greater than the carbon footprint associated with the average stenting procedure, at 2.4 tonnes CO_2_-e. Figure 2 presents a comparison of these two footprint values, including a breakdown by aggregated sector. In both procedures, a significant portion of the carbon footprint is generated by expenditure on goods and services that were not directly clinical in nature, such as utilities, non-medical furniture and equipment, and services (40% for CABG and 48% for stenting).

Of note, emissions associated with waste for both procedures were calculated as part of the expenditure on the Utilities sector, since SLHD accounting does not allocate the expenditure on waste based on the physical quantities of waste generated by each procedure. Expenditure on waste collection generated a total of 26.1 kg CO_2_-e for each CABG procedure, and 5.93 kg CO_2_-e for each stent procedure.

Figure 2 also illustrates the percentage contribution to the total carbon footprint for each procedure, based on the aggregated economic sector to which expenditure was allocated. The sectors contributing to these aggregates are given in the Supplementary materials. Figure 2 shows some interesting differences between the two procedures, with the largest contributor to the carbon footprint of the CABG procedure being expenditure on consumable supplies (36%), while the largest contributor to the carbon footprint of the stenting procedure was expenditure on other manufactured products within the supply chain (18%). A review of the cost bucket allocations for the two procedures confirms this difference, with the Ward and ED supplies cost bucket allocated 13.4% of the total expenditure for CABG, but only 2.7% of the total expenditure for stenting.

**Figure 3:**
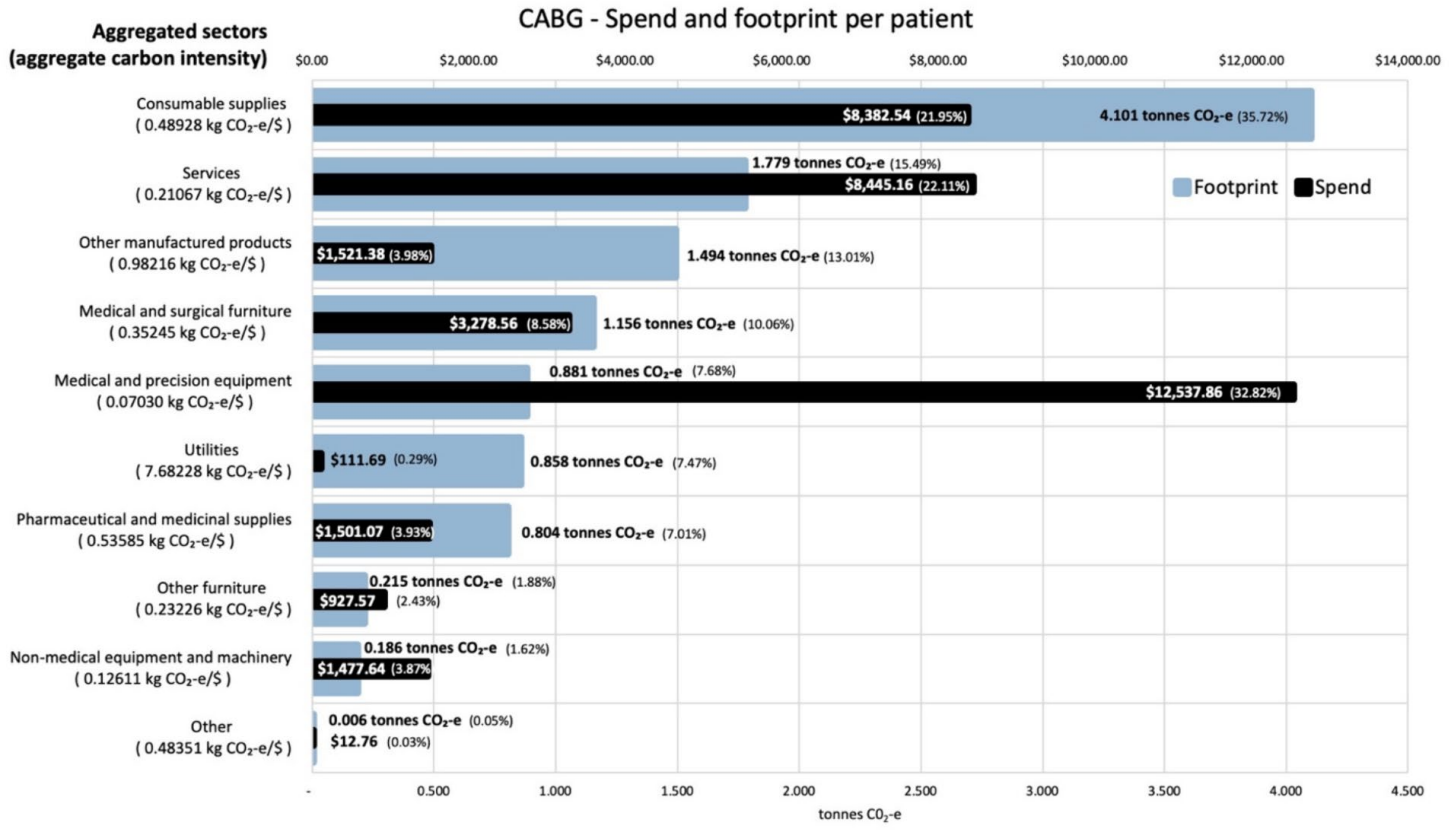
Carbon footprint contribution by weight and total spend in $ for each aggregated sector for activities associated with CABG procedures, per average patient. Carbon intensities for the aggregated sectors are given in the *Y* axis legend.

**Figure 4:**
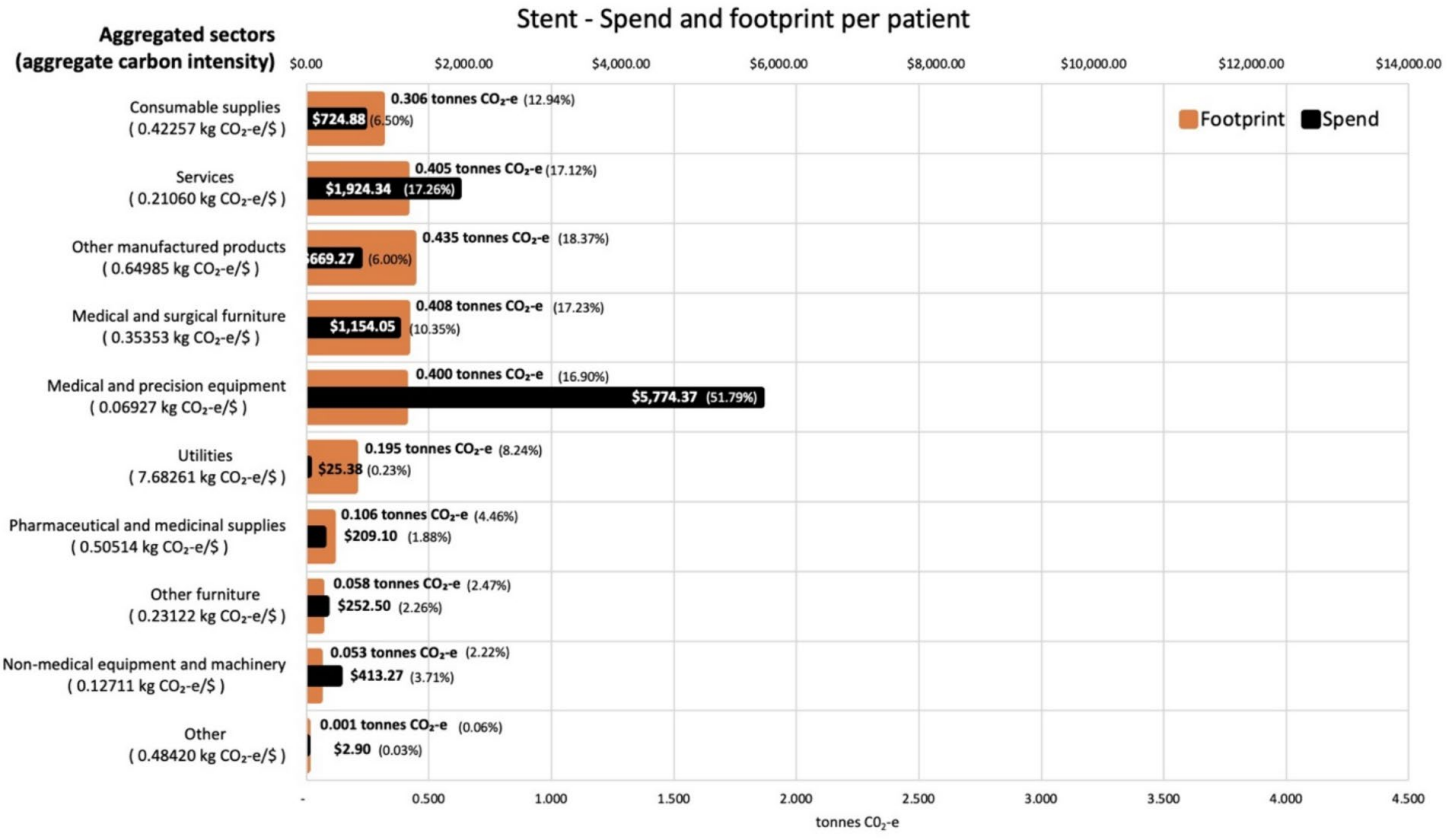
Carbon footprint contribution by weight and total spend in $ for each aggregated sector for activities associated with stent procedures, per average patient. Carbon intensities for the aggregated sectors are given in the *Y* axis legend.

Analysis of the contribution made by each sector to the total footprint reveals that the most carbon intensive sector was utilities, which contributes 7.7 kg CO_2_-e for every dollar spent on the sector across both procedures. This carbon intensity, along with that for the other aggregated sectors, is shown in Figure 3 for the activities associated with the CABG procedure. The carbon intensities for the activities associated with the stent procedure is shown in Figure 4. Pharmaceutical and other medicinal supplies was also a carbon intensive sector, contributing 0.54 kg CO_2_-e for every dollar spent on the sector for CABG patients and 0.51 kg CO_2_-e for every dollar spent on the sector for stent patients. The slight difference in intensity is due to the aggregated nature of the sectors presented, with a slightly different mix of contributing sub-sectors in each case. Supplementary materials show the carbon intensities for the contributing subsectors, these intensities have been derived from the EEIO model described in the methods section above. For the CABG procedure, costs allocated to consumable supplies influenced the total footprint value, contributing 4.1 tonnes per procedure (36% of the total). This included expenditure on supplies required to treat surgical wounds and was a relatively carbon intense sector, generating 0.49 kg CO_2_-e for every dollar spent on the sector. For the stenting procedure, expenditure allocated to prosthetics (27% of total), in the aggregated medical and precision equipment sector, has a less significant impact on the total footprint, contributing 0.2 tonnes per procedure (8% of the total), driven by a lower carbon intensity for the medical appliances sector, at 0.07 kg CO_2_-e for every dollar spent on the sector.

Our result equates to 0.14 kg CO_2_-e per dollar spent on the stenting and 0.16 kg CO_2_-e per dollar spent on the CABG, which aligns in scale with the sector wide intensity calculated by Malik et al. at 0.26 kg CO_2_-e per dollar spent (4). The lower carbon intensity of the cardiac activities studied, compared to the national health care sector intensity, may be attributable to the selection of uncomplicated patient care episodes, which we did to allow a direct comparison between stenting and CABG. To put this carbon intensity in context, cardiovascular disease accounted for 5.4% of the Australian burden of disease in 2019–20 and the clinical response accounted for 6.28% of all hospital costs in 2020–21 ($8.8 billion) (27). Our initial research suggests that the 48,000 stent and 12,700 CABG procedures conducted in Australia in 2020–21 as part of this clinical response, have an associated carbon footprint that is of commensurate scale (27). The results discussed here are based on an annual sample for one major Australian hospital. Further data sets from wider time periods and geographies would enhance the potential generalisability of these results. The timeframe considered in the study may not be entirely representative for a typical year, but it is likely that results prior or after COVID would be similar. Our results do demonstrate the application of the novel methodology proposed and provide a rich source of data for decision making within the studied context.

## 4 Discussion

Although there are unit process LCA studies of some of the individual aspects of patient care pathways (6, 12, 13), we believe that this is the first study to compare the carbon footprint of two common medical pathways adopted by clinicians to treat similar health conditions. The example of stable two vessel coronary disease was chosen *a priori*, as the clinical outcomes are generally very similar (19). We acknowledge that there may be subtle differences in post-procedure care over the subsequent years; up to 5–10% of stent patients might require re-intervention by 5 years, and there are some differences in medications (19). For example, stent patients more often require dual rather than single agent anti-platelet therapy for some months after the procedures; but CABG patients more often require anti-arrhythmic therapy. These can influence the comparative size of the systemic carbon footprints of these procedures, however these differences are likely to be very small, compared to the major peri-procedural differences informing the estimated carbon footprint (28). Given that, for these reasons, the choice of procedure is generally clinically equivalent, the choice between procedures exemplifies the type of instance where there would be a strong case for clinicians taking into account wider considerations such economic and environmental impact.

The magnitude of difference between the carbon footprints of each procedure quantified here, with the CABG footprint 4·9 times greater than the stent footprint, is to be expected to a certain extent, based on the relative difference in their costs, at 4·4 times. A more detailed review of the sector contributions reveals some additional insights however, with the much higher expenditure on ward and emergency department supplies associated with the CABG procedure influencing the relativity of these footprint values, effectively adding a carbon weighting to the difference in economic cost. The magnitude of difference between footprint results for these procedures indicates the potential contribution of environmental analysis, beyond clinical and economic considerations, in procedure selection.

Our results suggest that, in addition to the considerably lower financial cost associated with an elective stenting pathway, there was a considerably smaller systemic carbon footprint, when compared to a CABG pathway. Much of this reduced economic and environmental impact can be attributed to lesser requirements for post operative care. Usually, elective stenting will result in a post-operative overnight stay whereas post-operative care for CABG typically involves a 1–2 day stay in the Intensive Care Unit (ICU) and a further 5–7 day hospital ward stay (29, 30). Healthcare providers, patients, and health system administrators, seeking to work out the optimal treatment pathways, taking into consideration treatment outcomes, economic value, and a sectoral reduction in carbon emissions, could potentially draw on this evidence.

### 4.1 Systemic and specific carbon footprints

EEIO analysis can be understood as connecting sectoral GHG emissions to the expenditure that supports each procedure, which we refer to as a systemic carbon footprint. This can be contrasted with the now widespread use of unit process LCA to quantify emissions associated with health sector processes to assess the associated environmental impacts (6, 12, 13). To ensure a manageable analysis, the boundaries in LCA studies tend to be relatively confined, barring an analysis of the complex systems that support patient care pathways. By contrast, the use of EEIO to quantify the carbon footprint associated with these two cardiac procedures supports a boundaryless, systemic assessment of the total carbon emissions generated to support the delivery of these procedures at RPAH. These footprint results incorporate the whole of system emissions attributable to a procedure, including the emissions associated with operating the healthcare system and its facilities, infrastructure, and personnel, along with the apportioning of utilities, transport, and material inputs such as clinical supplies and medical devices and furniture.

The systemic scope of inclusion for EEIO analysis is highlighted in Table 2 when we compare our results with two other recent analysis of cardiac procedure carbon footprints. The 124.3 kg CO_2_-e emissions calculated by Grinberg et al. for conventional adult cardiac surgery in France, used an eco-audit approach (31). This simplified LCA approach may have impacted on the precision, robustness, and representativeness of their study (35). A subsequent LCA based estimate of the amount of CO_2_-e produced by CABG surgery at Tufts Medical Center was four times larger, at 505.1 kg CO_2_ − e per case (33). However, some of this difference may also be attributable to differences in the procedure, like Tuft’s requirement to autoclave all waste from cardiac suites, prior to disposal. Nonetheless both these results were well within the 6–814 kg CO_2_ − e range identified by Rizan et al. in their systematic review of surgical operation carbon footprints (36). The very significant difference between the footprint calculated by Grinberg et al. (31) and Hubert et al. (33), and our result of 11.5 tonnes CO_2_-e for the CABG procedure, suggests that a narrowly bounded focus on specific surgical or other clinical processes runs the risk of very significantly under-estimating the systemic carbon footprint of patient care pathways, by excluding the emissions associated with the operation of the health system and the comprehensive supply chains that support a particular procedure. For example, Grinberg et al. (31) only included disposable medical products, pharmaceutical products and electricity consumption used in anaesthesia, cardiopulmonary bypass and surgery. Similarly, Hubert et al. (33) estimated by collecting data from anaesthesia, electrical consumption, and generation of solid waste. They acknowledged that emissions from the heating, ventilation and air-conditioning (HVAC) system, reprocessing surgical instruments, pre and post-operative care were excluded. Neither study included a range of other sources of emissions in the patient encounter included in our study, including pathology, critical care, patient transport, as well as non-clinical sources.

**Table 2:** Comparison of three different CABG carbon footprint analysis.

Study	Method	Cohort	Scope (per procedure)	Data	Result (kg CO_2_ − e)
Grinberg et al., 2021 (31)	Eco-audit, using granular primary activity data. Single site study.	Single valve repair or replacement and isolated on-pump coronary artery bypass grafting in adults at Lyon University Hospital, France. (*n* = 28)	Surgical, anaesthesia and the cardiopulmonary bypass workstations. Disposable medical products, pharmaceutical products and electricity consumption.	Based on a bill of materials, process choice, transport requirements and duty cycle (the details of the energy and intensity of use), and disposal route. Considers the embodied energies and process energies from a database of material properties; those for the energy and carbon intensity of transport and the energy sources associated with use are drawn from look-up tables (32).	124.3
Hubert et al., 2022 (33)	LCA, using granular primary activity data. Single site study.	Uncomplicated CABG surgery for elective patients at Tufts Medical Center, USA (*n* = 18)	The surgical suite, defined as the sum of a hospital’s operating theatres, surrounding corridors, and sterile core, inclusive of anaesthetic and equipment rooms but exclusive of pre-operative and post-operative holding and recovery areas, administrative offices, and medical device reprocessing departments. Staff travel was excluded as it was considered outside of the study boundary (33).	Data were collected on volatile anaesthesia utilisation, electrical consumption, and generation of solid waste based on both trash bag collection and weights of the sharp container. Following GHG Protocol, considers scope 1, anaesthetic gases using Global Warming Potential (34); scope 2, electricity use (grid intensities provided by local electrical utilities), energy for space heating; and scope 3, surgical supply chain and waste disposal, applying DEFRA greenhouse gas life-cycle conversion factors for waste disposal, which take into account greenhouse gas emissions generated upstream in the supply chain as well as in the downstream disposal (33).	505.1
This study	EEIO, using averaged patient activity-based cost data. Single site study.	Coronary artery bypass using 1 LIMA graft and coronary artery bypass using 1 saphenous vein graft for stable, non-diabetic patients with two-vessel coronary disease at RPAH, Australia (*n* = 32).	Full patient encounter (admission to discharge). Operation of healthcare system and facilities, infrastructure, and personnel, along with the apportioning of utilities (including waste), transport, and material inputs such as clinical supplies and medical devices and furniture, excluding capital items.	Cost data for NHCDC for the financial year 2021–22 (Round 26) for costs incurred by Royal Prince Alfred Hospital (RPAH). Single-region input–output table using the Australian Industrial Ecology Laboratory for 1,284 sectors across the Australian economy, and the associated greenhouse gas emissions generated by each sector during the year, drawn from Australia’s National Greenhouse Inventory Report.	11,482

The difference between the results in Table 2 is superficially striking. However, these studies have quite substantially different scopes. The American and French LCA studies are designed consequentially, aiming to quantify the marginal additional climate change burden of the CABG procedures studied. By contrast our EEIO study can be understood as an attributional life cycle assessment, aiming to quantify the contribution of the patient care pathway associated with a CABG procedure in Australia to the overall burden of green-house gas emissions driving global heating. The difference between the footprint calculated by Grinberg et al. and Hubert et al. for conventional isolated cardiac procedures and our calculation for a CABG patient care pathway may, at least partially, be explained by these different scopes (31, 33). The scope of our calculations include the full range of activities contributing to each patients’ episode of care from admission to discharge. This includes allied health services, medical support, nursing, critical care, imaging, pathology, prosthetics, specialist procedures, ward and emergency department supplies, non-clinical and on-costs, and patient transport. Importantly, the pathway for a patient undergoing a CABG procedure typically involves an extended period of post operative care prior to discharge, consuming up to 9 days of hospital resources (28, 29). The consumption of these resources all contribute to the carbon footprint of a patient care pathway, but are not within scope for the LCA studies here discussed. All these essential aspects of delivering a cardiac procedure contribute to the health sector’s overall emissions, some 7% of Australia’s total emissions (4).

### 4.2 Potential applications of systemic footprints

In making recommendations to patients, healthcare providers have, to date, been almost exclusively influenced by the clinical outcomes of procedures. Increasingly, however, they are acknowledging that treatment choices also have economic and environmental consequences and, in the face of the climate crisis in particular, that environmental impacts also form an important component of healthcare decision making, including informing patients (37, 38). In this context, data about carbon footprinting of different procedures are essential to help healthcare providers understand the environmental impact of their clinical choices.

In this paper, we chose two procedures with relatively similar clinical outcomes, recognising that this is the circumstance where healthcare providers will be most interested in other impacts of their decisions, including those on the environment. There are many other examples in medicine where there are two or more choices for clinical care with similar outcomes, for example in the choice of asthma puffers (pressurised with propellants versus activated by inhalation), anaesthetic agents (intravenous versus volatile gases), bariatric surgery versus GLP-1 agonists for weight loss, and alternative forms of peritoneal dialysis. Such equipoise cases represent relatively uncontroversial entry points for guiding medical practitioners to consider differences in emissions profiles in their advice. Cases where emissions profiles are in tension with health outcomes will require far more public debate, but it can be anticipated that as the impacts of climate change become more severe, debates in health care will, as in other areas of public policy, need to more seriously consider climate impacts.

In addressing climate change impacts, healthcare planners will also benefit from a streamlined mapping of emissions hotspots across patient care pathways, made possible by the systemic carbon footprinting approach demonstrated in this paper. These hotspots can inform systems design for emerging primary care models in areas such as telehealth, introducing consideration of carbon emissions into analysis of health care delivery reform (39). Research in other sectors has shown that integrating activity-based costing evaluation and carbon footprint assessment can help managers incorporate environment costs into decision-making processes (40, 41). The method we describe may achieve similar results in informing health systems funding allocation, by providing planners with a carbon weighting to include in their activity-based funding determinations.

Operationally, hotspots identified through EEIO analysis will also constrain parameters for process-based LCA, allowing targeted collection of granular data to inform very specific LCA studies. These studies can be costly, but when targeted, can provide detailed guidance for refining facilities design and management, and for the refining clinical procedures, in areas such as waste management and alternative clinical technologies.

### 4.3 Sources of uncertainty in systemic footprinting

In their study of the carbon footprints of hospital care pathways Zhang et al. (14) conduct a data quality assessment of emissions factors and financial activity data like those used in this study. They found these to be ‘good’ or ‘very good’ in all aspects, although the framework they used is typically applied to physical data. Pertinently, recognising that Leontief’s basic input–output relationship cannot be differentiated analytically, Lenzen (42) describes a range of uncertainties associated with input/output analysis and compares these to truncation errors associated with standard unit process life cycle techniques. He notes that cumulatively, these uncertainties are smaller than the truncation errors for most commodities, and that this uncertainty decreases with the number of components in the assessment. We note that the number of components in our assessment of patient care pathways defies conventional unit-process assessment, suggesting a lower level of uncertainty. In a later paper Lenzen et al. (43) undertake a comprehensive uncertainty analysis to estimate standard deviations for carbon multipliers used in the calculation of the UK’s carbon footprint, using Monte Carlo techniques. They conclude that multipliers for consumer emissions exhibit relatively low Relative Standard Deviations of between 3.0 and 5.1%.

The contrast between the results of LCA studies of the carbon footprint of cardiac procedures and the EEIO study described here emphasizes the difference between these modelling approaches. Each has some limitations. Aside from the major difference in scope, limitations associated with these different methods may contribute to the contrasting scale of results. Unit process LCA, informed by detailed physical data, and EEIO, informed by financial activity data, are subject to different uncertainties (42). LCA studies typically suffer from some degree of truncation error, as they are unable to comprehensively map their entire supply chain. This has been estimated to lead to a more than 50% underestimate of emissions. Studies of more service based activities, such as those in healthcare, tend to have higher truncation errors (42). The degree to which more sophisticated LCA techniques address this limitation continues to be debated in the literature (44, 45). By contrast, EEIO provides a boundaryless and therefore comprehensive account of supply chain emissions, but is limited in the level of granularity that can be achieved given its comprehensiveness. The activity-based costing data used for estimating the footprint of an activity has been subject to several accounting transformations that may also contribute to uncertainty.

Bearing in mind both the different scopes and uncertainties associated with these distinctive modelling approaches, it is important to recognise that they are useful for different purposes: EEIO provides a comprehensive systemic footprint (which could inform strategic decision making such as health systems design and guidance on choice of patient care pathway), LCA provides a detailed specific footprint (which can inform tactical decision making). While unhindered by the need to select a boundary, limitations are introduced to any EEIO analysis through the sectoral resolution of the underlying input–output tables, providing far less granular data than the primary data collected for LCA studies. An EEIO analysis will not provide guidance on procurement decisions between alternate suppliers of goods and services, if they are in the same sector. It will also not provide detailed guidance for refining facilities design and management (for example optimising HVAC, or power standby) or for refining clinical care procedures (for example sterilisation and reuse rather than single use of clinical supplies). Further, specific to the health sector, some authors are cautious of distortions in costing, for example resulting from different drug pricing regimes, that may make it difficult to accurately connect different expenditure values to the resultant GHG emissions (13). Although the subsidies that potentially cause these distortions are incorporated into the input–output tables upon which EEIO analysis is based, these concerns do bear further investigation.

Other limitations of the methodology presented here are introduced with the high level of aggregation used in allocating the costs associated with each procedure to cost buckets, since these data need to be disaggregated to match the economic sectors present in the input–output table. This disaggregation process could be improved if more detailed insights were available on the allocation of detailed costs to each cost bucket, or if cost data were available at a finer resolution, such as at the cost centre level. Further studies using more highly disaggregated activity-based costing data are underway to understand the impact of this limitation.

Finally, the exclusion of depreciation costs is recognised as a limitation of the described method. This exclusion resulted in an underestimate of the systemic footprint, which did not include emissions associated with expenditure on capital items. This does warrant research into practical approaches to estimating capital systemic footprints. Further studies are underway, to explore the implications of these methodological differences and to establish guidelines for the contexts in which the different methodologies will be most appropriate.

## 5 Conclusion

Clinical guidance on the management of coronary disease has, to date, been silent on consideration of carbon footprints. As Tennison et al. comment in their carbon footprint assessment of the NHS, “The selection of a less carbon-intensive and resource intensive care practices where clinically appropriate can reduce both emissions and costs” (3 p.e90). Our results suggest that an elective stenting pathway has a substantially smaller systemic carbon footprint compared to a CABG pathway. Given clinical equipoise in the specific circumstance we have described, guidance on clinical decisions regarding treatment of stable patients with two-vessel coronary disease could consider both the economic and environmental costs, and thence recommend stenting.

This study demonstrates that clinical decision making can be informed by systemic carbon footprint analysis, to be considered alongside health care outcomes and economic indicators. In this way comparative estimates of systemic emissions attributable to different patient care pathways can contribute to the evidence base informing health care policy. EEIO analysis is a tool that is useful in this context because the activity based costing data required to conduct the assessment are readily available in Australia and many other jurisdictions (21), allowing cost effective and rapid analysis.

## Data Availability

The raw data supporting the conclusions of this article will be made available by the authors, without undue reservation.
