# Supplementary material for: Healthcare-related carbon footprinting—lower impact of a coronary stenting compared to a coronary surgery pathway
Source: Front Public Health. 2024 Aug 21;12:1386826. doi: 10.3389/fpubh.2024.1386826 (PMC11371610; doi:10.3389/fpubh.2024.1386826)
Supplement: Supplementary file 1 [file Table_1.pdf]

Supplementary information

1 1284 IOPC sectors and carbon intensities

IOPC (1284)	Sector name	Aggregated Sector	Intensity (kg/$)
1110010	Plants grown undercover	Agriculture, Forestry & Fishing	0.3901284
1120010	Plants grown outdoors	Agriculture, Forestry & Fishing	0.410664
1130010	Turf	Agriculture, Forestry & Fishing	0.3973987
1140010	Flowers (incl cut flowers) and flower seeds grown undercover	Agriculture, Forestry & Fishing	0.4169675
1150010	Flowers (incl cut flowers) and flower seeds grown outdoors	Agriculture, Forestry & Fishing	0.4351641
1210010	Mushrooms, fresh or chilled	Agriculture, Forestry & Fishing	0.4570168
1210020	Mushroom spawn	Agriculture, Forestry & Fishing	0.4371638
1220010	Lettuces grown undercover	Agriculture, Forestry & Fishing	0.4240577
1220020	Tomatoes grown undercover	Agriculture, Forestry & Fishing	0.436399
1220030	Other vegetables, fresh or chilled, grown undercover	Agriculture, Forestry & Fishing	1.021473
1230010	Lettuces grown outdoors	Agriculture, Forestry & Fishing	0.4460706
1230020	Tomatoes grown outdoors	Agriculture, Forestry & Fishing	0.4419184
1230030	Potatoes, sweet potatoes and edible roots and tubers nec grown outdoors	Agriculture, Forestry & Fishing	0.4465518
1230040	Beans, french and runner; peas, green or blue grown outdoors	Agriculture, Forestry & Fishing	0.4486977
1230050	Cabbages, brussels sprouts, cauliflowers and headed broccoli grown outdoors	Agriculture, Forestry & Fishing	0.4579606
1230060	Carrots grown outdoors	Agriculture, Forestry & Fishing	0.5198726
1230070	Onions grown outdoors	Agriculture, Forestry & Fishing	0.4863641
1230080	Vegetable seeds	Agriculture, Forestry & Fishing	0.4771636
1230090	Other vegetables (incl. melons), fresh or chilled grown outdoors	Agriculture, Forestry & Fishing	0.4874517
1310010	Grapes - table	Agriculture, Forestry & Fishing	1.907568
1310020	Grapes - wine	Agriculture, Forestry & Fishing	0.3403186
1310030	Grapes sun-dried or for drying	Agriculture, Forestry & Fishing	0.4680143
1320010	Kiwi fruit	Agriculture, Forestry & Fishing	0.4284148
1330010	Strawberries	Agriculture, Forestry & Fishing	0.4797627
1330020	Berries nec - fresh and sun-dried	Agriculture, Forestry & Fishing	0.4171722

IOPC (1284)	Sector name	Aggregated Sector	Intensity (kg/$)
1340010	Apples - fresh and sun-dried	Agriculture, Forestry & Fishing	0.4582002
1340020	Pears and quinces - fresh and sun-dried	Agriculture, Forestry & Fishing	0.457883
1350010	Stone fruit - fresh and sun-dried	Agriculture, Forestry & Fishing	0.50804
1360010	Citrus fruit - fresh and sun-dried	Agriculture, Forestry & Fishing	0.6859533
1370010	Olives - fresh and sun-dried	Agriculture, Forestry & Fishing	0.3907203
1390010	Bananas - fresh and sun-dried	Agriculture, Forestry & Fishing	0.4545869
1390020	Orchard fruit nec - fresh and sun-dried	Agriculture, Forestry & Fishing	0.4789472
1390030	Almonds and macadamias	Agriculture, Forestry & Fishing	0.9426503
1390040	Edible nuts (excluding Peanuts) nec; Other fruit nec - fresh and sun-dried	Agriculture, Forestry & Fishing	0.6674384
1400010	Sheep and lambs	Agriculture, Forestry & Fishing	2.026744
1400020	Wool (shorn and dead)	Agriculture, Forestry & Fishing	6.433502
1400030	Sheep and lamb products nec	Agriculture, Forestry & Fishing	1.876864
1400040	Sheep and beef cattle agistment services	Agriculture, Forestry & Fishing	1.937413
1400050	Beef cattle and calves	Agriculture, Forestry & Fishing	2.1465
1400060	Beef cattle and calves products (excluding Milk) nec	Agriculture, Forestry & Fishing	1.987882
1400070	Rice, in the husk	Agriculture, Forestry & Fishing	1.687729
1400080	Wheat (incl spelt) and meslin, unmilled	Agriculture, Forestry & Fishing	3.897544
1400090	Barley, unmilled	Agriculture, Forestry & Fishing	2.712664
1400100	Oats, unmilled	Agriculture, Forestry & Fishing	2.286669
1400110	Grain, sorghum	Agriculture, Forestry & Fishing	2.035278
1400120	Lupins (white or yellow) for grain	Agriculture, Forestry & Fishing	11.61807
1400130	Oilseeds	Agriculture, Forestry & Fishing	11.65062
1400140	Legumes for grain nec	Agriculture, Forestry & Fishing	1.745402
1400150	Cereal grains nec	Agriculture, Forestry & Fishing	1.954365
1510010	Sugar cane (for planting or crushing)	Agriculture, Forestry & Fishing	0.416368
1520010	Cotton (excl ginned)	Agriculture, Forestry & Fishing	0.2612115
1590010	Forage sorghum	Agriculture, Forestry & Fishing	0.4623157
1590020	Forage products nec	Agriculture, Forestry & Fishing	0.7377239
1590030	Tobacco	Agriculture, Forestry & Fishing	122.8439
1590040	Beverage and spice crops	Agriculture, Forestry & Fishing	0.6068344
1590050	Grass, lucerne and clover seed	Agriculture, Forestry & Fishing	0.5022574
1590060	Hay, cereal grasses and fodder	Agriculture, Forestry & Fishing	0.4562136
1590090	Peanuts	Agriculture, Forestry & Fishing	0.5089062
1590110	Natural rubber	Agriculture, Forestry & Fishing	0.5509912
1590120	Crops nec	Agriculture, Forestry & Fishing	0.4017543

IOPC (1284)	Sector name	Aggregated Sector	Intensity (kg/$)
1600010	Whole milk, chilled but otherwise untreated	Agriculture, Forestry & Fishing	1.789863
1600020	Dairy cattle	Agriculture, Forestry & Fishing	3.111693
1710010	Poultry, for slaughtering	Agriculture, Forestry & Fishing	0.589391
1720010	Eggs	Agriculture, Forestry & Fishing	0.6477271
1720020	Egg laying hens	Agriculture, Forestry & Fishing	0.6081401
1800010	Deer	Agriculture, Forestry & Fishing	0.5947686
1910010	Thoroughbred horses	Agriculture, Forestry & Fishing	0.6543348
1910020	Horse stud and breeding services; horses nec	Agriculture, Forestry & Fishing	0.6483563
1910030	Horse Agistment Services	Agriculture, Forestry & Fishing	0.6795418
1920010	Pigs	Agriculture, Forestry & Fishing	0.5995645
1930010	Unblended honey and beeswax	Agriculture, Forestry & Fishing	0.8503454
1990010	Pet breeding and live animals nec	Agriculture, Forestry & Fishing	0.6505253
1990020	Livestock products nec	Agriculture, Forestry & Fishing	0.6090375
2000010	Farmed oysters (including Pearl), paua and molluscs nec	Agriculture, Forestry & Fishing	0.2671303
2000020	Farmed seaweed	Agriculture, Forestry & Fishing	0.2454433
2000030	Farmed fish and fish hatchery products	Agriculture, Forestry & Fishing	0.268509
2000040	Farmed prawns and crustaceans nec	Agriculture, Forestry & Fishing	0.2520298
2031980	General government consumption of fixed capital (0201-0203)	Other	122.9487
3010010	Natural gums and resins (incl oleoresins)	Agriculture, Forestry & Fishing	0.2344844
3010020	Softwoods (conifers) growing	Agriculture, Forestry & Fishing	0.2455384
3010030	Hardwoods, brushwoods, scrubwoods - growing	Agriculture, Forestry & Fishing	0.2653103
3010040	Forest products nec	Agriculture, Forestry & Fishing	0.2346497
3020010	Softwoods (conifers) logs	Agriculture, Forestry & Fishing	0.2732087
3020020	Hardwoods, brushwoods, scrubwoods - logs; hewn timber and timber nec (incl firewood)	Agriculture, Forestry & Fishing	0.2347411
3021980	General government consumption of fixed capital (0301-0302)	Other	0.2313026
4110010	Rock lobster and crab	Agriculture, Forestry & Fishing	0.5054359
4120010	Prawns	Agriculture, Forestry & Fishing	0.2427374
4130010	Fish and squid (line fishing)	Agriculture, Forestry & Fishing	0.2988348
4140010	Fish (trawling or netting)	Agriculture, Forestry & Fishing	0.263293
4190010	Oysters and other aquatic invertebrates nec, live, fresh or chilled	Agriculture, Forestry & Fishing	0.2753429
4190020	Coral and similar, shells of molluscs; natural animal sponges; algae, fresh or dried	Agriculture, Forestry & Fishing	0.4493611

IOPC (1284)	Sector name	Aggregated Sector	Intensity (kg/$)
4190030	Freshwater fish and aquatic animals nec	Agriculture, Forestry & Fishing	0.2412683
4200010	Skins and pieces, raw	Agriculture, Forestry & Fishing	0.2418808
4200020	Wildlife culling services	Services	0.2274861
4200030	Hunting and trapping products nec	Agriculture, Forestry & Fishing	0.2012673
4201980	General government consumption of fixed capital (0411-0420)	Other	0.2277452
5100010	Services to forestry nec	Services	0.2914667
5210010	Cotton (ginned), cotton seed, waste from cotton and cotton ginning services	Agriculture, Forestry & Fishing	3.727919
5220010	Sheep shearing services	Services	0.2964324
5290010	Aerial agricultural services	Services	0.2968032
5290020	Services to agriculture nec	Services	0.2969624
5290030	Services to fishing nec	Services	0.2933171
5290040	Wool classing services	Services	0.2988395
5291980	General government consumption of fixed capital (0510-0529)	Other	0.2979079
6000010	Black coal (all types incl briquettes)	Mining	9.893492
6000020	Brown coal-lignite (incl briquettes)	Mining	0.7789122
7000010	Crude oil (incl. condensate)	Mining	0.868584
7000020	Liquefied natural gas	Mining	9.753042
7000030	Natural gas (in the gaseous state)	Mining	0.3703906
7000040	Coal gas, water gas, producer gas and similar gases (excl petroleum gases and other gaseous hydrocarbons)	Mining	0.4693589
8010010	Iron ore (incl treatment; excl pelletising)	Mining	10.83925
8010020	Agglomerated iron ores nec (incl iron ore pellets and sintered ores)	Mining	0.6257947
8020010	Bauxite	Mining	0.6485976
8030010	Copper concentrates, oxides and ores	Mining	1.164804
8040010	Gold bullion and ores	Mining	0.4748452
8050010	Beneficiated ilmenite, ilmenite and leucoxene concentrates	Mining	0.5366743
8050020	Rutile concentrates	Mining	2.596654
8050030	Monazite, xenotime and zircon concentrates; mineral sand ores nec	Mining	3.015339
8060010	Nickel ores and concentrates	Mining	0.5418418

IOPC (1284)	Sector name	Aggregated Sector	Intensity (kg/$)
8070010	Lead ores and concentrates (excl silver-lead-zinc ores)	Mining	1.02861
8070020	Silver and zinc ores	Mining	0.7408254
8090010	Tin, tin-copper and tin-tantalite concentrates	Mining	0.7010189
8090020	Uranium concentrates	Mining	16.40061
8090030	Manganese ores	Mining	1.431123
8090040	Non-ferrous metallic ores and concentrates nec (incl tungsten)	Mining	7.660237
9110010	Gravel	Mining	0.7129824
9110020	Sand	Mining	0.7070185
9190010	Dimension stone	Mining	0.8755783
9190020	Pebbles, broken or crushed stone, macadam, tarred macadam, granules, chippings and powder of stone	Mining	0.6484082
9190030	Limestone (incl shell and coral)	Mining	0.7099018
9190040	Clays nec (incl brick, pipe, tile and shale)	Mining	0.7260531
9190050	Construction materials (mined) nec (incl decomposed rock, residues, etc) (excl crushed and broken stone and dimension stone)	Mining	3.16682
9900010	Salt	Mining	0.6981029
9900020	Precious and semi-precious gemstones (incl garnet concentrate)	Mining	1.175635
9900030	Gypsum; anhydrite; calcareous stone of a kind used for the manufacture of lime or cement (excluding limestone)	Mining	0.9820558
9900040	Silica	Mining	2.071525
9900050	Natural phosphates and Phosphate rock (unground)	Mining	0.7054765
9900060	Chemical and fertilizer minerals nec	Mining	0.6846127
9900070	Non-metallic minerals nec	Mining	8.659008
10110010	Petroleum exploration	Mining	0.532463
10120010	Mineral exploration	Mining	0.502412
10900010	Mining services nec	Services	0.5038031
10901980	General government consumption of fixed capital (1011-1090)	Other	0.4995795
11110010	Fresh meat, chilled or frozen (excl kangaroo or horse meat, other than for human consumption)	Other	4.838681

IOPC (1284)	Sector name	Aggregated Sector	Intensity (kg/$)
11110020	Fresh kangaroo or horse meat, other than for human consumption	Other	1.647148
11110030	Casings, bungs, weasands and runners (incl gut materials for further processing)	Agriculture, Forestry & Fishing	3.271276
11110040	Edible offals (excl poultry offals)	Other	6.791529
11110050	Edible tallow (excl refined)	Other	1.473327
11110060	Inedible tallow (excl refined) and other unrefined animal oils and fats	Agriculture, Forestry & Fishing	108.3703
11110070	Raw hides and skins	Agriculture, Forestry & Fishing	1.388199
11110080	Meat (excl fresh) for human consumption	Other	1.424852
11110090	Blood meal (milled or screened dried blood) for use as stock or poultry feed	Agriculture, Forestry & Fishing	1.370204
11110100	Inedible meat or meat offal flours, meals and pellets; greaves	Agriculture, Forestry & Fishing	5.143923
11110110	Other animal products nec	Agriculture, Forestry & Fishing	4.346483
11120010	Poultry and poultry products (incl canned)	Other	1.334934
11130010	Bacon and ham and other dried, salted or smoked pigmeat (incl canned)	Other	1.221354
11130020	Smallgoods nec (incl crumbed lamb cutlets, cured meat (canned or uncanned), frankfurters, saveloys and salami)	Other	1.357102
11131700	Meat and meat products - commission production (1111-1113)	Other	1.391464
11200010	Fish, canned	Other	0.14831
11200020	Frozen whole fish, fish fillets and fish meat; fish loaf, cake, balls and paste; smoked fish; fish fingers; caviar	Other	0.1543655
11200030	Rock lobster and crayfish (incl tails), chilled or frozen (incl boiled and frozen)	Other	0.1960501
11200040	Crustaceans, molluscs & aquatic invertebrates nec (chilled, frozen, preserved or otherwise prepared)	Other	0.1656484
11200050	Inedible flours, meals, pellets & other products nec of fish, crustaceans & molluscs or other aquatic invertebrates	Other	0.133173
11201700	Processed seafood - commission production	Other	0.1314251

IOPC (1284)	Sector name	Aggregated Sector	Intensity (kg/$)
11201980	General government consumption of fixed capital (1120)	Other	122.9965
11310010	Processed liquid milk (incl whole milk and skim)	Other	1.39372
11310020	Cream (incl thickened), not concentrated or sweetened	Other	1.322952
11320010	Ice cream and frozen confections	Other	1.246266
11330010	Flavoured whole milk drinks	Other	1.241058
11330020	Sour cream, yoghurt and other cultured milk products	Other	1.180045
11330030	Buttermilk	Other	1.353158
11330040	Fats and oils derived from milk (incl butter oil); casein	Other	1.153905
11330050	Butter	Other	1.194225
11330060	Cheese and curd	Other	1.564086
11330070	Milk based food preparations (excluding malt extracts) and dried milk based mixes	Other	1.384503
11330080	Milk and cream, concentrated or sweetened; lactose and lactose syrup; products of natural milk constituents nec	Other	1.457003
11331700	Dairy products - commission production (1131-1133)	Other	1.019078
11400010	Jams	Other	0.4492579
11400020	Fruit juices, single strength or concentrated	Other	0.414691
11400030	Dried fruit (excl sun-dried)	Other	0.4747244
11400040	Preserved fruit and fruit products nec	Other	0.5167241
11400050	Vegetables, frozen	Other	0.4197549
11400060	Vegetables, prepared or preserved (incl dried or shelled)(excl frozen); pickles and chutney	Other	1.196431
11400070	Tomato pulp, puree and paste	Other	0.4619373
11400080	Mixed meat and vegetables, canned	Other	0.4831924
11400090	Vegetable juices (incl mixtures)(incl tomato); mixtures of vegetable and fruit juices	Other	0.3736406
11400100	Pasta products, canned	Other	0.4132561
11400110	Soup and homogenised food preparations including fruit, vegetables, meat or composites thereof	Other	0.6080195
11400120	Sauces (excl worcestershire and apple); vinegar (excl wine vinegar)	Other	0.5155304

IOPC (1284)	Sector name	Aggregated Sector	Intensity (kg/$)
11400130	Fresh vegetable salads, in plastic containers	Other	0.4808912
11400140	Fruit and vegetable based health, invalid or baby preparations	Other	0.5951124
11400150	Dried roots, tubers and vegetables; Flour and meal of vegetables nec.	Other	0.3559638
11401700	Fruit and vegetable products - commission production	Other	0.3517551
11500010	Crude soya bean, cotton seed, peanut, sunflower, safflower, rape seed, coconut and vegetable oils	Other	1.196253
11500020	Refined and processed animal or vegetable oils and fats (incl tallow) (excl neatsfoot, wool grease and lanolin)	Other	1.453684
11500030	Margarine	Other	1.243805
11500040	Oil-cake, acid oils, cotton linters and other solid residues resulting from the extraction or refining of vegetable fats or oils	Other	1.106704
11501700	Oils and fats - commission production	Other	0.9736387
11610010	Wheat and other cereal flours (incl self-raising)	Other	1.261723
11610020	Flour mill products nec, for human consumption	Other	1.428673
11610030	Wheat bran for human consumption (excl for breakfast food)	Other	1.85932
11610040	Starch of wheat and corn	Other	1.655474
11610050	Glucose, glucose syrup (incl dextrose) and modified starches (incl dextrins)	Other	1.273517
11610060	Wheat gluten and tapioca	Other	2.01079
11610070	Prepared baking powders	Other	1.313652
11610080	Malt (excl malt extract)	Other	8.131379
11610090	Malt extract	Other	2.375092
11610100	Rice (husked, semi-milled or wholly milled)	Other	1.775738
11610110	Rice groats, meals and pellets; other worked cereal grains and products nec;	Other	1.80509
11620010	Cereal foods (incl breakfast foods)	Other	1.744238
11620020	Mixes and doughs nec (incl custard powder) for preparation of bakers wares (excl frozen)	Other	1.292491
11620030	Pasta	Other	2.399801
11621700	Grain mill and cereal products - commission production (1161-1162)	Other	131.7251

IOPC (1284)	Sector name	Aggregated Sector	Intensity (kg/$)
11700010	Bread and bread rolls	Other	0.8920372
11700020	Biscuits and biscuit crumbs; rusks; ice cream cones and wafers; unleavened bread	Other	0.9278626
11700030	Cakes, pastries and crumpets	Other	0.9104199
11700040	Meat pies	Other	0.8227862
11700050	Biscuit and bread dough (incl frozen)	Other	1.273949
11701700	Bakery products - commission production (1171-1174)	Other	0.7552349
11810010	Raw and refined sugar in solid form (incl brown sugar)(excl icing sugar)	Other	1.516723
11810020	Icing sugar, molasses (incl treacle) and sugar nec	Other	0.9019256
11810030	Liquid refined sugar, golden syrup, artificial honey, starch and sugar products nec	Other	1.109849
11820010	Chocolate confectionery (excl chocolate coated biscuits and white chocolate)	Other	1.024984
11820020	Cocoa beans (roasted); cocoa paste, powder, butter, fat or oil	Other	0.8524495
11820030	Other food preparations containing cocoa (excl chocolate confectionery)	Other	1.179218
11820040	Chewing gum, white chocolate and other confectionery not containing cocoa	Other	1.058533
11820050	Crystallised, drained and glace fruit, nuts and peel	Other	1.000841
11821700	Sugar and confectionery - commission production (1181-1182)	Other	0.3707072
11910010	Potato crisps and flakes	Other	0.6212181
11910020	Corn chips; taco, tortilla and tostada shells	Other	0.6163552
11920010	Dog and cat food (excl canned)	Other	0.6462268
11920020	Dog and cat food, canned	Other	0.6536432
11920030	Bran, sharps and other residues (excl rice, wheat and rye), for animal feed	Other	0.5190605
11920040	Prepared animal and bird feeds nec (incl poultry pellets, crumbles and mash)	Other	0.5391944
11920050	Cereal groats, meals, pellets and other cereal products nec, other than for human consumption	Other	0.5847201
11990010	Coffee and tea, including substitutes	Other	0.6019181
11990020	Yeast and yeast extracts	Other	0.5936108
11990030	Nuts, roasted	Other	0.6718178

IOPC (1284)	Sector name	Aggregated Sector	Intensity (kg/$)
11990040	Spices	Other	0.6401134
11990050	Mustard; worcestershire sauce; mayonnaise and salad dressing	Other	0.7651941
11990060	Flavouring essences, industrial	Other	0.527869
11990070	Prepared meals (incl TV dinners), of meat or meat offal	Other	0.6770266
11990080	Bakers' wares nec (incl pretzels and frozen pizza) (excl bread and pies)	Other	0.6144438
11990090	Peanut butter and other nut butters, pastes and purees; jams	Other	0.1223706
11990100	Refined salt (cooking and table)	Other	1.975594
11990110	Gelatine	Other	0.5871884
11990120	Food products nec (incl jelly crystals, meat pastes)	Other	0.7390473
11991700	Other food products - commission production (1191-1199)	Other	95.89498
11991970	Waste from the manufacture of food, food products and beverages (excl alcohol) (1111-1211)	Other	0.5621287
11991980	General government consumption of fixed capital (1191-1199)	Other	122.9431
12110010	Natural and artificial mineral waters and aerated waters (excl sweetened or flavoured)	Other	0.6503919
12110020	Natural water nec	Other	0.5831343
12110030	Mineral waters and aerated waters, sweetened or flavoured, canned	Other	0.682589
12110040	Mineral waters and aerated waters, sweetened or flavoured, bottled	Other	0.6672933
12110050	Cordials and syrups; powder flavours for soft drinks; concentrated cordial extracts	Other	1.069227
12110060	Sweetened or flavoured bulk pre-mix & post-mix concentrates for mineral & aerated waters; non-alcoholic beverages nec	Other	0.6621787
12110070	Ice	Other	0.4953911
12111700	Soft drinks, cordials and syrups - commission production	Other	0.3292891
12120010	Beer, ale and stout, bottled	Other	1.313045
12120020	Beer, ale and stout, canned	Other	1.486557
12120030	Beer, ale and stout, bulk	Other	1.275097

IOPC (1284)	Sector name	Aggregated Sector	Intensity (kg/$)
12121700	Beer - commission production	Other	0.3879772
12130010	Whisky, brandy, rum, gin and fortified spirits; other distilled alcoholic beverages (incl liqueurs and mixed drinks)	Other	0.8496925
12130020	Vermouth and distillation wine	Other	0.5905488
12140010	Wines (incl sparkling) of grapes and other fruit (excl vermouth)	Other	1.067224
12140020	Cider, perry, mead and wine-based mixed drinks (coolers)	Other	0.6063171
12140030	Vinegar from wine	Other	0.4224098
12141700	Wine, spirits and other alcoholic beverages - commission production (1213-1214)	Other	0.410057
12141970	Waste from the manufacture of alcohol (1212-1214)	Other	0.4033082
12200010	Cigarettes, cigars, cheroots and tobacco	Other	0.4825526
12201700	Cigarette and tobacco products - commission production	Other	122.0516
12201970	Waste from the manufacture of tobacco products	Other	122.9972
13110010	Wool, scoured or carbonised (degreased but not carded or combed)	Consumable supplies	6.482297
13110020	Wool tops; noils of wool or fine animal hair	Consumable supplies	1.070402
13110030	Wool grease and fatty substances derived from wool grease (incl lanolin)	Consumable supplies	4.453169
13120010	Yarn and thread (excl chenille, loop wale, elastic or elastomeric) of natural fibres (incl worsted) nec	Consumable supplies	0.8844506
13120020	Chenille, loop wale yarn and thread of natural fibres	Consumable supplies	0.9259749
13120030	Natural textile fibres prepared for spinning nec	Consumable supplies	1.017802
13120040	Narrow woven textile fabrics (incl tape) (excl bias binding) of natural fibres; tyre cord fabric of high tenacity yarn of natural fibres	Consumable supplies	0.8746621
13120050	Woven and broadwoven pile, chenille and terry fabrics of cotton or other natural fibre (excl narrow fabrics)	Consumable supplies	1.014928
13120060	Woven and broadwoven fabrics of natural fibres, other than cotton, nec (excl pile, chenille and terry)	Consumable supplies	1.001949

IOPC (1284)	Sector name	Aggregated Sector	Intensity (kg/$)
13120070	Woven and broadwoven fabrics of cotton (excl pile, chenille and terry)	Consumable supplies	0.9993009
13130010	Textured, high tenacity, single, elastomeric, synthetic or artificial yarns and threads nec	Consumable supplies	1.006926
13130020	Gimped yarn and strip; chenille and loop-wade yarn; woven or broadwoven fabrics of metal thread and metallised yarn nec	Consumable supplies	0.8494658
13130030	Elastomeric yarn of cotton, wool or fine animal hair (containing polyurethane or similar thread, excl rubber thread)	Consumable supplies	0.5064782
13130040	Yarn of glass fibre	Consumable supplies	0.01856294
13130050	Woven or broadwoven fabric of artificial or synthetic filaments and fibres (excl pile or chenille)	Consumable supplies	1.024858
13130060	Woven pile fabrics and chenille fabrics (other than narrow fabrics) of man-made fibres	Consumable supplies	0.8885238
13130070	Narrow woven textile fabrics (incl tape) (excl bias binding) of synthetic fibres; tyre cord fabric of high tenacity yarn of synthetic fibres	Consumable supplies	1.129278
13131700	Textiles - commission production (1311-1313)	Consumable supplies	0.9984096
13200010	Leather, vegetable or chrome tanned (incl re-tanned), dressed or finished; chamois leathers	Consumable supplies	0.3940688
13200020	Leather (excl dressed or finished)	Consumable supplies	0.2927511
13200030	Raw hides and skins, pickled or otherwise preserved	Consumable supplies	4.525011
13200040	Tanned or dressed skins, with hair or wool retained (incl sheepskin rugs)	Consumable supplies	0.2921685
13200050	Handbags, suitcases, bags, travel sets for personal toilet articles, purses, key cases, wallets and billfolds (excl paper)	Consumable supplies	0.2991402
13200060	Saddlery and harness, of any material; leather articles nec	Consumable supplies	0.3265183
13200070	Rucksacks of leather or leather substitute	Consumable supplies	0.04218068
13201700	Tanned leather, dressed fur and leather products - commission production	Consumable supplies	0.04496321
13310010	Carpets and other textile floor coverings (incl mats and matting) (excl felt and underfelt)	Consumable supplies	0.694445

IOPC (1284)	Sector name	Aggregated Sector	Intensity (kg/$)
13320010	Rope and cable (excl wire), cordage (excl tyre cord yarn), twine or net products	Consumable supplies	0.7148297
13330010	Textile interior furnishing articles (incl blankets (excl electric), wall coverings, curtains, bed and table linen nec)	Consumable supplies	0.717183
13330020	Towels (incl tea towels) and face washers of cotton terry towelling or similar cotton terry fabrics	Consumable supplies	0.4313992
13330030	Baby napkins of textile fabrics	Consumable supplies	0.6707559
13330040	Textile quilted prods, hose/tubing, nonwovens, (bonded & yarn fabrics)	Consumable supplies	0.7386227
13330050	Curtains in the piece (incl continuous), knitted or crocheted	Consumable supplies	0.7323185
13330060	Textile tarpaulins (incl canvas), sails, tents, annexes, pneumatic mattresses and motor vehicle covers	Consumable supplies	0.7058543
13330070	Blinds and awnings of textile fabrics (incl canvas) and woven textile materials (incl cotton)	Consumable supplies	0.7349985
13330080	Bags, sacks and packets of textile or canvas	Consumable supplies	0.6856373
13330090	Textile motor vehicle seat covers	Consumable supplies	0.7358997
13330100	Pillows, cushions, bolsters, bean bags and stuffed mattress protectors (excl those of or stuffed with rubber)	Consumable supplies	0.703433
13330110	Floor-cloths, dishcloths, dusters and similar cleaning cloths	Consumable supplies	0.7011716
13330120	Textile life jackets, life-belts, sleeping bags, parachutes and other cut and sewn textile products nec	Consumable supplies	0.8427805
13340010	Labels and badges with printed or woven lettering or design	Consumable supplies	0.7058459
13340020	Textile finishing nec	Consumable supplies	0.7185608
13340030	Felt floor coverings (exclude underfelt)	Consumable supplies	0.7432203
13340040	Underfelt and other felt products (excl floor coverings, headwear or clothing)	Consumable supplies	0.727626
13340050	Textile fabrics (excl rubber or plastic coated) & articles (excl bags) of a kind commonly used in machinery or plant	Consumable supplies	1.76806

IOPC (1284)	Sector name	Aggregated Sector	Intensity (kg/$)
13340060	Wadding, powder puffs, pads, cotton wool, gauze and bandages	Pharmaceutical and medicinal supplies	0.7208998
13340080	Articles of bonded fibre or yarn fabrics (excl labels & badges); tapestries, textile sutures, transmission and conveyor belts, textile articles nec	Consumable supplies	0.4659135
13340090	Braids, tassels, tulles; lace or embroidery, strips or motifs	Consumable supplies	0.706779
13340100	Special fabrics nec	Consumable supplies	0.003823358
13340110	Garment dyeing service	Services	0.5511141
13341700	Textile products - commission production (1331-1334)	Consumable supplies	0.658664
13341980	General government consumption of fixed capital (1331-1334)	Other	122.9802
13400010	Hosiery (incl pantyhose, stockings, tights and socks)	Consumable supplies	0.05099674
13400020	Pullovers, jumpers, sweaters and cardigans - knitted	Consumable supplies	0.04300788
13400030	Knitted or crocheted pile fabrics (excl elastic or elastomeric)	Consumable supplies	0.06004652
13400040	Knitted or crocheted fabric nec	Consumable supplies	0.04125897
13400050	Knitted products nec	Consumable supplies	0.05552298
13401700	Knitted products - commission production	Consumable supplies	122.995
13510010	Mens and boys trousers (excl suits), shorts, jeans, overalls and work shirts, dustcoats, textile (excl waterproof)	Consumable supplies	0.05722563
13510020	Men's & boys' suits or uniforms (incl trousers for suits & uniforms), coats & jackets, textile (excl waterproof)	Consumable supplies	0.05905589
13510030	Women's and girls' dresses, skirts, slacks, shorts, tunics, uniforms, jeans, overalls, leotards, coats, capes, suits and ensembles	Consumable supplies	0.06054205
13510040	Shirts and blouses (with collars)	Consumable supplies	0.05736147
13510050	T-shirts and tank tops	Consumable supplies	0.05523506
13510060	Swimwear; sweatsuits, tracksuits, jogging suits, leisure suits and jumpsuits	Consumable supplies	0.05894891
13510070	Foundation garments (incl brassieres, corsets and girdles)	Consumable supplies	0.05131648

IOPC (1284)	Sector name	Aggregated Sector	Intensity (kg/$)
13510080	Underwear	Consumable supplies	0.05116488
13510090	Outer nightwear (incl dressing gowns and robes) and sleepwear	Consumable supplies	0.03229735
13510100	Waterproof, plastic or rubber trousers, overalls, coats and jackets	Consumable supplies	0.0561907
13510110	Plastic (unsupported film) clothing other than waterproof	Consumable supplies	0.04699181
13510120	Wetsuits and other rubber clothing and accessories nec (incl gloves, belts)(excl headgear)	Consumable supplies	0.04897114
13510130	Fur and sheepskin clothing and clothing accessories (excl headwear, footwear, handbags, purses and toys)	Consumable supplies	0.06699513
13510140	Hats and other headgear (excl safety, rubber or plastic)	Consumable supplies	0.04352934
13510150	Safety headgear; textile belts for clothing; plastic clothing accessories (excl belts and disposable gloves)	Consumable supplies	0.04250549
13510160	Safety eyewear (industrial or sporting)(incl goggles)	Consumable supplies	0.04845869
13510170	Clothing and clothing accessories nec	Consumable supplies	0.05107259
13511700	Clothing - commission production	Consumable supplies	0.04496581
13520010	Footwear with uppers and outer soles of rubber or plastic (incl waterproof footwear and thongs) (excl sports footwear)	Consumable supplies	0.09132997
13520020	Footwear with uppers of leather and outer soles of rubber or plastic (excl sports footwear)	Consumable supplies	0.09577214
13520030	Sports footwear	Consumable supplies	0.08961367
13520040	Footwear with uppers of leather and outer soles of leather or composition leather (excl sports footwear)	Consumable supplies	0.1020465
13520050	Footwear nec (incl steel capped footwear)	Consumable supplies	0.11231
13520060	Soles of or cut from rubber or rubber composition and parts of footwear nec (incl plastic heels)	Consumable supplies	0.08746938
13521700	Footwear - commission production	Consumable supplies	122.9958
13521970	Waste from the manufacture of textiles, clothing or footwear (1311-1352)	Consumable supplies	0.1682489

IOPC (1284)	Sector name	Aggregated Sector	Intensity (kg/$)
14110010	Undressed sawn timber from logs sawn at same establishment (incl treated (excl impregnated sleepers or resawn)); shooks	Consumable supplies	0.2958656
14110020	Treated wood in the rough (excl sawn timber, dressed or undressed); impregnated railway sleepers	Consumable supplies	0.2853426
14110030	Ground bark	Consumable supplies	0.3034713
14120010	Woodchips, softwood	Consumable supplies	0.5361874
14120020	Woodchips, hardwood	Consumable supplies	1.1873
14130010	Resawn/seasoned timber (incl kiln dried)(excl sleepers, palings & shingles)	Consumable supplies	0.2939339
14130020	Dressed timber and mouldings of a thickness up to and including 6mm	Consumable supplies	0.2956275
14130030	Dressed timber and mouldings of a thickness exceeding 6mm	Consumable supplies	0.2871578
14130040	Chemically preserved re-sawn or dressed timber.	Consumable supplies	0.2993467
14131700	Drying, impregnation or chemical treatment - commission production (1411-1413)	Consumable supplies	0.2914341
14910010	Prefabricated or transportable wooden buildings	Consumable supplies	0.2884125
14920010	Doors, wooden	Consumable supplies	0.2823107
14920020	Roof trusses, wooden	Consumable supplies	0.2864602
14920030	Wooden wall and window (incl complete with glass) frames	Consumable supplies	0.2793322
14920040	Custom made built-in wooden furniture	Consumable supplies	0.2786799
14920050	Other wooden builders joinery and carpentry	Consumable supplies	0.2778516
14930010	Veneers (incl laminated)	Consumable supplies	0.3012314
14930020	Plywood	Consumable supplies	0.2833263
14930030	Glued laminated lumber	Consumable supplies	0.2759276
14940010	Fibreboard (excl fibre paperboard and particle board)	Consumable supplies	0.2886427
14940020	Cellular wood panels	Consumable supplies	0.2783018
14940030	Particle board (incl laminated) and similar board of wood or other ligneous materials	Consumable supplies	0.276043
14940040	Laminates of timber and non-timber materials	Consumable supplies	0.2867064
14940050	Other boards manufactured from wood nec (incl densified wood in block or other shape)	Consumable supplies	0.2898234

IOPC (1284)	Sector name	Aggregated Sector	Intensity (kg/$)
14990010	Parquetry strips etc., assembled into panels; shingles and shakes	Consumable supplies	0.2862345
14990020	Pallets, cases, boxes, crates, drums, casks and barrels, wooden	Consumable supplies	0.2750311
14990030	Frames, wooden (incl for paintings, photographs, mirrors, etc)	Consumable supplies	0.2746683
14990040	Boards & similar articles nec, of vegetable fibre agglomerated with mineral binders (excl wooden boards)	Consumable supplies	0.2974716
14990050	Moulding boxes, patterns, bases; moulds for metal (excl ingot), glass, mineral materials, rubber or plastics	Consumable supplies	0.3005192
14990060	Wooden tools, tool bodies & handles; cork articles (incl agglomerated)(excl gaskets for motor vehicles); other products of wood nec	Consumable supplies	0.2772819
14991700	Other wood products - commission production (1491-1499)	Consumable supplies	0.2762442
15100010	Mechanical, chemical and semi-chemical wood pulp and residual lyes from wood pulp (excl tall oil)	Consumable supplies	0.8199263
15100020	Newsprint	Consumable supplies	0.8499942
15100030	Paper stock (incl toilet, facial tissue and similar paper stock used for household or sanitary purposes)	Consumable supplies	0.8198681
15100040	Copying paper nec	Consumable supplies	1.111551
15100050	Paper and paperboard, coated, impregnated, covered, surface-coloured, surface-decorated nec	Consumable supplies	1.069415
15100060	Paper and paperboard, uncoated nec	Consumable supplies	1.198566
15101700	Paper - commission production	Consumable supplies	0.8612863
15210010	Solid and corrugated paperboard containers	Consumable supplies	0.5076364
15210020	Corrugated paperboard sheeting	Consumable supplies	0.5226374
15220010	Paper bags, packets and sacks (incl paper multiwall bags) (excl bags of composite material)	Consumable supplies	0.6658321
15230010	Envelopes (paper), letter & correspondence cards (excl printed or illustrated); paper wallets & writing compendiums of paper	Consumable supplies	0.616613

IOPC (1284)	Sector name	Aggregated Sector	Intensity (kg/$)
15230020	Exercise books, registers, account books, diaries, board games and other paper stationery (excl commission printing)	Consumable supplies	0.608743
15240010	Toilet, tissues, serviettes, towels & similar paper for household & sanitary purposes, in sheets or perforated rolls	Consumable supplies	0.5857647
15240020	Baby napkins (excl textile), sanitary towels and tampons of paper or cellulose wadding	Consumable supplies	0.5899131
15290010	Paper and paperboard trays, dishes, plates, cups, cones, egg containers and box files	Consumable supplies	0.5398913
15290020	Paper festival, carnival or other entertainment articles (incl conjuring tricks, novelties, Christmas decorations)	Consumable supplies	0.5491694
15290030	Adhesive paper labels (excl printed)	Consumable supplies	0.003726712
15290040	Other paper, paper pulp or paperboard products (incl wallpaper and liquid activated gummed or adhesive paper)	Consumable supplies	0.4345793
15291700	Paper products - commission production (1521-1529)	Consumable supplies	0.5255207
15291970	Waste from manufacture of wood and paper products (1411-1529)	Consumable supplies	1.156677
15291980	General government consumption of fixed capital (1521-1529)	Other	122.9283
16110010	Books (incl atlases & touring guides), maps, charts, plans, sheet music printed but not published by this business	Consumable supplies	0.0284227
16110020	Newspapers, journals and periodicals printed but not published by this business once a week or more	Consumable supplies	0.4667419
16110030	Newspapers, journals and periodicals printed but not published by this business less than weekly	Consumable supplies	0.1588223
16110040	Security printed material (incl stamps, cheque books, banknotes, share documents and airline tickets)	Consumable supplies	0.585968
16110050	Paper labels, printed or imprinted (but not published)	Consumable supplies	0.523641
16110060	Letter and correspondence cards (printed but not published), postcards	Consumable supplies	0.5669706

IOPC (1284)	Sector name	Aggregated Sector	Intensity (kg/$)
16110070	Trade advertising material or commercial catalogues printed but not published by this business; other printed matter nec	Consumable supplies	0.5724914
16120010	Composed type, prepared printing plates/cylinders, lithographic stones or other impressed media for use in printing	Consumable supplies	0.9358896
16120020	Printing trade services nec (excluding desktop publishing)	Services	0.4734009
16200010	Pre-recorded Audio and Video tapes, manufactured but not published by this business	Other manufactured products	0.3984346
16200020	Reproduced computer software, manufactured but not published by this business	Other manufactured products	0.6532463
16200030	Pre-recorded Audio CD's and DVD's, manufactured but not published by this business	Other manufactured products	0.7103247
16200040	Other pre-recorded media (including records), manufactured but not published by this business	Other manufactured products	1.18216
16201980	General government consumption of fixed capital (1611-1620)	Other	123.1907
17010010	Automotive petrol; gasoline refining or blending; motor spirit (incl aviation spirit)	Other manufactured products	0.3923239
17010020	Kerosene (incl kerosene type jet fuel)	Other manufactured products	0.4877372
17010030	Gas oil or fuel oil (excl motor spirit and kerosene)	Other manufactured products	0.4553862
17010040	Petroleum bitumen; residues of petroleum oils and bituminous minerals; petroleum coke	Other manufactured products	0.4459244
17010050	Liquefied petroleum gas produced at refineries	Other manufactured products	0.5429938
17010060	Lubricating, heavy petroleum & bituminous oils; solvents; topped/enriched crude, refinery products nec	Other manufactured products	0.2339518
17090010	Metallurgical coke, coke breeze, retort carbon and char (excl bone char)	Other manufactured products	0.5593217
17090020	Pitch and pitch coke obtained from mineral tars	Other manufactured products	0.3773654
17090030	Mineral turpentine	Other manufactured products	0.401749
17090040	Petroleum jelly; paraffin wax and other mineral waxes	Consumable supplies	0.5376638
17090050	Toluol, xylol, not chemically or commercially pure; benzole and benzene from petroleum	Other manufactured products	0.5373867
17090060	Phenol	Other manufactured products	0.5282671

IOPC (1284)	Sector name	Aggregated Sector	Intensity (kg/$)
17090070	Styrene	Other manufactured products	0.3866076
17090080	Chloroform and other halomethanes	Other manufactured products	0.4776877
17090090	Carbon tetrachloride	Other manufactured products	0.000764093
17090100	Brake and hydraulic fluid	Other manufactured products	0.4855483
17090110	Rust arresting compound	Other manufactured products	0.5619567
17090120	Bituminous mixtures and other articles of asphalt	Other manufactured products	0.417505
17090130	Petroleum and coal products nec.	Other manufactured products	0.5905971
17091700	Petroleum and coal products - commission production (1701-1709)	Other manufactured products	0.391191
17091970	Waste from the manufacture of Petroleum and Coal products (1701-1709)	Other manufactured products	0.3740855
18110010	Acetylene gas	Other manufactured products	0.5620388
18110020	Hydrogen, rare gases, nitrogen, medicinal gases (incl nitrous oxide and oxygen), carbon dioxide (incl dry ice) and carbon monoxide	Pharmaceutical and medicinal supplies	0.489905
18110030	Ethylene gas	Other manufactured products	0.5693
18110040	Liquefied natural gas (other than from the well head)	Other manufactured products	25.19512
18110050	Hydrogen sulphide, Sulphur dioxide and other industrial organic and inorganic gases nec	Other manufactured products	27.32003
18120010	Carbon black	Other manufactured products	0.6191815
18120020	Synthetic organic colouring agents & preparations (incl colour lakes, pigments & dyes of vegetable or animal origin)	Other manufactured products	0.4985369
18120030	Hydrocarbons and derivatives (incl ethane, betane and benzole and benzene other than from petroleum and iron and steel)	Other manufactured products	0.5708725
18120040	Nitrogen-function compounds (excl saccharin)	Other manufactured products	0.5632004
18120050	Organo-inorganic compounds; heterocyclic compounds; nucleic acids	Other manufactured products	0.5673493
18120060	Carboxylic, monocarboxylic & polycarboxylic acids and derivatives (excl pharmaceutical goods)	Other manufactured products	1.311446
18120070	Ethyl alcohol pure	Consumable supplies	0.4984531
18120080	Other alcohols, phenols (excl phenol), phenol-alcohols and derivatives; fatty acids (purity less than 90%)	Consumable supplies	0.1293486

IOPC (1284)	Sector name	Aggregated Sector	Intensity (kg/$)
18120090	Plasticiser; mixed alkylbenzenes and alkylnaphthalenes nec; other chemical products and preparations nec	Other manufactured products	0.5512908
18120100	Ethers, alcohol peroxides, ether peroxides, epoxides, acetals and hemiacetals and derivatives; organic chemicals nec	Other manufactured products	0.5593017
18130010	Hydrochloric, chlorosulphuric, sulphuric (incl oleum), diphosphorous pentaoxide, phosphoric, and polyphosphoric acids	Other manufactured products	0.5576352
18130020	Nitric, sulphonitric and other inorganic acids; inorganic oxygen compounds of non-metals (excl industrial gases)	Other manufactured products	0.5430983
18130030	Synthetic inorganic colouring agents and preparations (incl inorganic pigments and chemical whites)	Other manufactured products	2.217723
18130040	Refined salt other than cooking or table salt	Other manufactured products	1.111788
18130050	Triammonium phosphate; ammonia (excl fertiliser); ammonium chloride & carbonates; potassium nitrate	Other manufactured products	3.450784
18130060	Prepared pigments, opacifiers, colours, glazes used in ceramic, enamel, glass industry; glass powder, granules/flakes	Other manufactured products	0.5814591
18130070	Artificial graphite; colloidal or semi-colloidal graphite; preparations based on carbon in form of semi-manufactures	Other manufactured products	0.7607828
18130080	Radioactive elements, nuclear reactor fuel elements, isotopes and compounds; alloys, dispersions, ceramic products and mixtures	Consumable supplies	1.152009
18130090	Other inorganic chemicals nec	Other manufactured products	1.079597
18210010	Synthetic rubber	Other manufactured products	0.5624649
18210020	Polystyrene	Other manufactured products	0.5368608
18210030	Polyethylene	Other manufactured products	0.8920688
18210040	Polyvinyl chloride	Other manufactured products	0.5319585
18210050	Polypropylene	Other manufactured products	0.622262
18210060	Polyvinyl acetate & synthetic resins nec (excl adhesives) in primary forms, not mixed/compounded (excl regranulated)	Other manufactured products	0.5697853

IOPC (1284)	Sector name	Aggregated Sector	Intensity (kg/$)
18210070	Rosin and resin acids, and derivatives thereof, rosin spirit and rosin oils; run gums	Other manufactured products	0.5657288
18210080	Plastics in primary forms, mixed/compounded with other substances; regranulated, single thermoplastic scrap material	Other manufactured products	0.5593288
18290010	Cellulose fibre or filament	Other manufactured products	130.9859
18290020	Non-cellulose fibre or filament	Other manufactured products	131.9406
18290030	Synthetic fibre or filament nec	Other manufactured products	0.5615844
18290040	Basic polymers nec	Other manufactured products	0.5419227
18310010	Ammonia aqua or urea, fertiliser grade; ammonium sulphate	Other manufactured products	0.554759
18310020	Superphosphate and other phosphatic fertilisers	Other manufactured products	0.5370926
18310030	Ammonium nitrate (excl explosive)	Other manufactured products	0.6821586
18310040	Mixed fertilisers	Other manufactured products	0.6430038
18310050	Ground phosphate	Other manufactured products	0.4944644
18310060	Fertilisers nec	Other manufactured products	0.5583217
18320010	Insecticides, pesticides, fungicides, weedkillers and pest control chemicals nec	Other manufactured products	0.5708458
18321700	Basic Chemicals - commission production (1811-1832)	Other manufactured products	0.5820897
18321970	Waste from the manufacture of basic chemicals (1811-1832)	Other manufactured products	0.4191944
18410010	Pharmaceutical goods, for human use (excl wadding, gauze, bandages and surgical sutures)	Pharmaceutical and medicinal supplies	0.2677511
18411700	Human Pharmaceutical and Medicinal Products - commission production	Pharmaceutical and medicinal supplies	108.6185
18420010	Animal feed supplements	Other manufactured products	0.0479595
18420020	Pharmaceutical goods for veterinary use	Other manufactured products	0.04675706
18421700	Veterinary Pharmaceutical and Medicinal Products - commission production	Other manufactured products	39.50202
18421970	Waste from the manufacture of pharmaceutical goods for human or veterinary use (1841-1842)	Pharmaceutical and medicinal supplies	122.9803
18510010	Glycerol (glycerine), glycerol waters and lyes	Other manufactured products	0.2896899
18510020	Candles and tapers	Other manufactured products	0.290496
18510030	Soap and soap based products	Consumable supplies	0.2932632
18510040	Toothpaste and other dentifrices	Consumable supplies	0.2175275
18510050	Laundry bleach	Consumable supplies	0.3848872

IOPC (1284)	Sector name	Aggregated Sector	Intensity (kg/$)
18510060	Disinfectants (incl phenyl)	Consumable supplies	0.2651484
18510070	Anionic, cationic and other organic surface active agents (excl soap)	Consumable supplies	0.3851323
18510080	Scouring preparations and abrasive cleaners	Consumable supplies	0.3095879
18510090	Surface cleaning, washing and degreasing preparations nec (incl oven and stove cleaners)	Consumable supplies	0.3514929
18510100	Other cleaning polishes, creams and waxes nec	Consumable supplies	0.3364588
18520010	Barrier creams and toilet lanolin; sunscreening preparations	Consumable supplies	0.3257774
18520020	Hair shampoo, conditioner, sprays, colouring and other hairdressing preparations	Consumable supplies	0.3908196
18520030	Aftershave & shaving preparations; lipstick, eye makeup; beauty cream or lotions; face lotions & powders	Consumable supplies	0.3860041
18520040	Hand cream or lotions (excl barrier & medicated cream); nail polishes & other nail care preparations	Consumable supplies	0.4249827
18520050	Perfume, deodorants, bath salts, depilatories, talcum powder and other preparations nec	Consumable supplies	0.364413
18521700	Cleaning Compounds and Toiletry Preparations - commission production (1851-1852)	Consumable supplies	0.2898357
18521970	Waste from the manufacture of cleaning compounds and toiletry preparations (1851 -1852)	Consumable supplies	122.9801
18910010	Photographic, film, cloth, plates (sensitised), photographic chemicals and photographic paper (sensitised)	Other manufactured products	0.7386116
18920010	Safety fuses, detonating fuses or caps	Other manufactured products	0.2415416
18920020	Explosives and other pyrotechnic articles (incl Ammonium nitrate (explosive), nitrocellulose, gun cotton, signalling flares, fireworks and matches)	Other manufactured products	0.525078
18990010	Eucalyptus, sandalwood and Tea-tree oil	Other manufactured products	0.6542196
18990020	Natural gums (processed or refined)	Other manufactured products	0.5479954
18990030	Fluxes and other preparations (incl pickling preparations, powders and pastes) for soldering, brazing or welding	Other manufactured products	0.4984134
18990040	Other chemical products nec	Other manufactured products	0.6615052

IOPC (1284)	Sector name	Aggregated Sector	Intensity (kg/$)
18991700	Other Basic Chemical Products - commission production (1891-1899)	Other manufactured products	132.3712
18991970	Waste from the manufacture of other basic chemical products (1891-1899)	Other manufactured products	122.98
18991980	General government consumption of fixed capital (1891-1899)	Other	122.98
19110010	Self-adhesive plastic plates, film, foil, tape, strip and other flat shapes	Other manufactured products	0.2309802
19110020	Flexible plastic strip, plates, film, foil, tape and sheet (excl self-adhesive)	Other manufactured products	0.196825
19110030	Plastic-coated, pressure-sensitive, gummed or adhesive paper and paperboard	Other manufactured products	0.2563169
19110040	Plastic sacks, packets and bags (incl garbage bags)	Consumable supplies	0.2428373
19110050	Textile fabrics (excl tyre cord) impregnated, coated, covered or laminated with plastics	Consumable supplies	0.2408853
19120010	Plastic bottles	Consumable supplies	0.2517356
19120020	Plastic table and kitchenware (incl disposable cups), other household (incl buckets) and toilet articles	Consumable supplies	0.2838131
19120030	Complete and assembled other domestic furniture (plastic only) not elsewhere specified	Other furniture	3.14196E-05
19120040	Other complete and assembled non-domestic furniture (plastic only) nec	Other furniture	0.2297998
19120050	Other medical, dental, surgical or veterinary furniture and parts (plastic only)	Medical and surgical furniture	0.2561911
19120060	Unassembled or partly assembled domestic furniture and parts (plastic only) nec	Other furniture	0.242343
19120070	Unassembled or partly assembled non-domestic furniture and parts (plastic only) nec	Other furniture	0.2337239
19120080	Plastic pipes	Consumable supplies	0.2491912
19120090	Plastic fittings for tubes, pipes and hoses (incl joints, elbows and flanges)	Consumable supplies	0.2730275
19120100	Plastic taps, cocks, valves and similar attachments	Consumable supplies	0.2475901
19120110	Plastic drums, drum linings, boxes, cases, crates & packaging accessories. (incl stoppers, lids, caps & seals)	Consumable supplies	0.232913
19120120	Polycarbonate sheets	Other manufactured products	0.2519193

IOPC (1284)	Sector name	Aggregated Sector	Intensity (kg/$)
19120130	Plastic blow moulded products nec	Consumable supplies	0.2555379
19120140	Other rigid or semi-rigid plastic injection moulded products (excl toys, games and fibre reinforced products)	Consumable supplies	0.238746
19120150	Rigid and semi-rigid polymer products nec (excl fibre reinforced plastic products)	Consumable supplies	0.2547517
19130010	Foam and sponge plastic sheets, plates and strip (incl foam insulation and padding)	Consumable supplies	0.2377928
19130020	Plastic foam products nec	Consumable supplies	0.2484112
19140010	New pneumatic, rubber tyres for motor cars and motor cycles	Other manufactured products	0.2360287
19140020	New pneumatic, rubber tyres for buses and lorries	Other manufactured products	0.001687027
19140030	Tyres (solid rubber)	Other manufactured products	0.1898664
19140040	Tyres, rubber nec (incl retreaded tyres)	Other manufactured products	0.2561336
19140050	Pneumatic rubber tubes	Other manufactured products	0.304566
19140060	Camel-back and unvulcanised rubber strip for retreading rubber tyres	Other manufactured products	0.08397658
19150010	Adhesives (excl bituminous) and glues	Other manufactured products	0.259966
19160010	Architectural & decorative paints (incl coatings for use on buildings), enamels & clears (excl heavy duty coatings)	Other manufactured products	0.2318679
19160020	Automotive paints (incl primer & undercoats), enamels, lacquers (excl heavy duty coatings & bituminous mastics)	Other manufactured products	0.4336295
19160030	Industrial paints (incl primer, undercoats, finishing coats and heavy duty coats), enamels and clears	Other manufactured products	0.2376479
19160040	Inks	Other manufactured products	0.2512869
19160050	Filler or putty, caulking compound	Other manufactured products	0.2563624
19160060	Other paints (incl marine coatings) and other allied products (incl thinners, wood stains, paint, rubbing compounds and varnish remover)	Other manufactured products	0.2625337
19190010	Plastic tubes and hoses	Consumable supplies	0.2982578
19190020	Plastic conveyor belting	Other manufactured products	0.2636343
19190030	Plastic wall or ceiling coverings (excl tiles)	Other manufactured products	0.2328892
19190040	Linoleum and other floor coverings with a textile base; plastic floor coverings (incl paper or paperboard base), wall or ceiling tiles	Other manufactured products	0.2525415

IOPC (1284)	Sector name	Aggregated Sector	Intensity (kg/$)
19190060	Rigid fibre reinforced plastic articles (incl swimming pool shells and tanks)	Other manufactured products	0.2525733
19190070	Other plastic injection moulded products nec (excl rigid or semi-rigid)	Consumable supplies	0.2356874
19190080	Artificial guts (sausage casing) of hardened protein or of cellulosic materials	Other manufactured products	0.409114
19190090	Synthetic rubber products and other polymer products nec (excl rigid or semi-rigid)	Consumable supplies	0.273335
19191700	Polymer Products - commission production (1911-1919)	Other manufactured products	0.2438572
19200010	Rubber gloves, mittens and mitts	Consumable supplies	0.35343
19200020	Rubber belting (incl V belts)	Other manufactured products	0.3337259
19200030	Rubber tubes, pipes and hose	Consumable supplies	0.29541
19200040	Rubber sheets, strips, plates, rods, profile shapes and primary forms (excl cellular)	Other manufactured products	0.3369688
19200050	Sponge and foam rubber	Consumable supplies	0.3042978
19200060	Other natural rubber products nec	Other manufactured products	0.2912829
19201700	Natural Rubber Products - commission production	Other manufactured products	131.7354
19201970	Waste from manufacture of polymer products, rubber, natural rubber and rubber products (1911-1920)	Other manufactured products	0.2845135
20100010	Float, surface ground/polished glass, in sheets; cast & rolled glass, in sheets or profiles; but not otherwise worked	Other manufactured products	0.8854185
20100020	Safety glass (incl windscreens and laminated sheet glass)	Other manufactured products	0.8864115
20100030	Glass containers, bottles or jars; glass stoppers; glass inners for vacuum vessels	Consumable supplies	0.898611
20100040	Rear-view mirrors for vehicles	Other manufactured products	0.000502158
20100050	Glassware nec	Consumable supplies	0.9315879
20101700	Glass and glass products - commission production	Consumable supplies	0.8528134
20210010	Clay bricks (excl refractory bricks)	Other manufactured products	0.2899541
20290010	Refractory products (incl bricks, cement and clay)	Other manufactured products	0.3077305
20290020	Ceramic roofing, flooring and wall tiles (incl terracotta) and ceramic construction goods nec	Other manufactured products	0.3069674
20290030	Ceramic wash basins and permanent fixture type sanitary ware	Consumable supplies	0.3094487

IOPC (1284)	Sector name	Aggregated Sector	Intensity (kg/$)
20290040	Tableware, ornamental pottery and domestic ware nec	Consumable supplies	0.3390012
20290050	Ceramic goods nec	Other manufactured products	0.3433215
20291700	Ceramic products - commission production (2021-2029)	Other manufactured products	122.7716
20310010	Cement (incl hydraulic and portland) (excl adhesive or refractory)	Other manufactured products	0.7247445
20310020	Lime (incl hydraulic, quick, hydrated, slaked and agricultural)	Other manufactured products	0.7664367
20320010	Plaster boards, sheets, panels, tiles, cornices and other articles of plaster (excl ornamental)	Other manufactured products	0.7714487
20320020	Plasters (incl plaster of paris)(excl dental plasters)	Consumable supplies	0.8319034
20330010	Ready mixed concrete and mortar (incl dry mix concrete)	Other manufactured products	0.753821
20331700	Cement, lime and ready-mixed concrete - commission production (2031, 2033)	Other manufactured products	0.7654381
20340010	Concrete, cement, fibrous-cement or artificial stone pipes; concrete box culverts	Other manufactured products	0.8040848
20340020	Concrete, cement and artificial stone bricks, blocks, building boards and tiles	Other manufactured products	0.7482784
20340030	Concrete or predominantly concrete prefabricated and transportable buildings	Other manufactured products	0.7836462
20341700	Plaster and concrete products - commission production (2032, 2034)	Other manufactured products	0.3505099
20900010	Worked monumental or building stone	Other manufactured products	0.3144141
20900020	Glass fibre and glass wool products	Other manufactured products	0.3185806
20900030	Ground limestone	Other manufactured products	0.2916691
20900040	Ground clays (excl colours); andalusite, kyanite & sillimanite; mullite; chamotte & dinas earths	Other manufactured products	0.5048278
20900050	Ground mica; feldspar; leucite; nepheline ; ground natural abrasives; crushed, powdered natural steatite and talc	Other manufactured products	0.986932
20900060	Ground minerals & fluorspar (excl abrasives, dust & powders of natural & synthetic precious or semi-precious stones)	Other manufactured products	0.6443818

IOPC (1284)	Sector name	Aggregated Sector	Intensity (kg/$)
20900070	Non-refractory mortars and concretes other than ready mixed; articles of asbestos-cement and cellulose fibre-cement nec	Other manufactured products	0.4040971
20900080	Other non-metallic mineral products	Other manufactured products	0.3144819
20901700	Other non-metallic mineral products - commission production	Other manufactured products	122.9262
20901970	Waste from the manufacture of non-metallic mineral products (2010-2090)	Other manufactured products	0.7509837
21100010	Basic iron, pig iron, sponge iron and spiegeleisen; iron or steel granules and powders	Other manufactured products	1.733085
21100020	Ferro-alloys (incl manganese, silicon or chrome)	Other manufactured products	2.146675
21100030	Iron or steel primary forms (incl ingots) and semi-finished products	Other manufactured products	1.746739
21100040	Iron or non-alloy steel flat-rolled products (excl clad, plated or coated)	Other manufactured products	3.273814
21100050	Clad, plated or coated iron or non-alloy steel flat-rolled products	Other manufactured products	1.866591
21100060	Alloy steel flat-rolled products	Other manufactured products	1.800367
21100070	Iron and steel bars, rods, angles, shapes and sections (incl sheet piling)	Other manufactured products	1.790907
21100080	Iron or steel wire for further processing (excl fencing, stranded or barbed)	Other manufactured products	1.870149
21100090	Painted, varnished or coated steel sheet, profile decking or cladding (incl steel sheeting for fencing)	Other manufactured products	1.753439
21100100	Iron or steel rails, rail fastenings or other rail accessories	Other manufactured products	2.02223
21100110	Iron or steel expanded metal	Other manufactured products	1.76447
21100120	Light oils obtained as a by-product from metallurgical coke (excl grease oils, toluole and xylole); Benzole from iron and steel manufacturing	Other manufactured products	4610.207
21100130	Crude tar	Other manufactured products	22724.99
21100140	Gas from coke works or blast furnaces	Other manufactured products	3019.811
21210010	Cast iron tubes, pipes and hollow profiles; cast iron or cast steel tube or pipe fittings	Other manufactured products	1.932059
21210020	Cast iron or cast steel steam, gas and water fittings other than domestic (incl taps, cocks and valves)	Other manufactured products	1.808451
21210030	Cast articles of iron or steel nec	Other manufactured products	1.672709

IOPC (1284)	Sector name	Aggregated Sector	Intensity (kg/$)
21220010	Iron or steel seamless tubes or pipes (excl cast or forged)	Other manufactured products	1.841158
21220020	Iron or steel tubes, pipes, hollow profiles and fittings (excl cast iron or seamless)	Other manufactured products	1.810395
21220030	Steel steam, gas and water fittings other than domestic (incl taps, cocks and valves)(excl cast steel)	Other manufactured products	1.948885
21221700	Iron and steel - commission production (2110-2122)	Other manufactured products	1.752249
21221970	Waste from manufacture of iron and steel (incl slag, dross, sealings and scrap steel) (2110-2122)	Other manufactured products	32.8393
21310010	Alumina	Other manufactured products	9.698656
21320010	Aluminium and aluminium alloys (excl purchased scrap)	Other manufactured products	14.52803
21320020	Aluminium secondary recovery from purchased scrap	Other manufactured products	9.495968
21320030	Aluminium castings and diecastings	Other manufactured products	0.9652837
21321970	Aluminium scrap from the manufacture of alumina, aluminium and aluminium alloys (2131-2132)	Other manufactured products	122.9878
21330010	Silver primary and secondary recovery (excl from purchased scrap)	Other manufactured products	1.07366
21330020	Copper (including brass) primary and secondary recovery (excl from purchased scrap)	Other manufactured products	1.126536
21330030	Lead primary and secondary recovery (excl from purchased scrap)	Other manufactured products	15.71765
21330040	Zinc primary and secondary recovery (excl from purchased scrap)	Other manufactured products	20.36585
21330050	Silver, copper (including brass), lead and zinc recovery from purchased scrap	Other manufactured products	7.216973
21330060	Zinc alloys; copper matte; cement copper; unwrought copper and nickel	Other manufactured products	7.438767
21330070	Sulphuric acid from the smelting of copper, silver, lead and zinc.	Other manufactured products	1.037784
21390010	Platinum primary and secondary recovery (excl from purchased scrap)	Other manufactured products	1.448806

IOPC (1284)	Sector name	Aggregated Sector	Intensity (kg/$)
21390020	Nickel and tin primary recovery and secondary recovery from drosses, ashes or other waste materials (excl from purchased scrap)	Other manufactured products	27.27407
21390030	Nickel and tin recovery from purchased scrap	Other manufactured products	2.839848
21390040	Gold - primary and secondary (excl from purchased scrap)	Other manufactured products	49.41049
21390050	Antimony and other non-ferrous basic metals nec primary and secondary recovery	Other manufactured products	5.864061
21390060	Basic precious metals (excl silver) secondary recovery from purchased scrap	Other manufactured products	1.075225
21390070	Other non-ferrous metal alloys	Other manufactured products	3.638309
21391700	Basic non-ferrous metals - commission production (2131-2139)	Other manufactured products	1.207378
21391970	Wastes and scraps from the smelting and refining of non-ferrous metals (incl precious) (2133-2139)	Other manufactured products	5.472709
21410010	Non-ferrous metal (excl aluminium) castings and diecastings	Other manufactured products	1.073609
21420010	Aluminium and aluminium alloy bars, rods (incl wire rod) and profiles (incl decking and cladding)	Other manufactured products	1.077117
21420020	Aluminium foil	Other manufactured products	1.090659
21420030	Rolled, drawn or extruded aluminium pipes, tubes, plates, sheets, strip & wire products; aluminium powders & flakes	Other manufactured products	1.13876
21490010	Copper, copper alloy, nickel, lead, zinc and tin rolled, extruded and semi-finished products	Other manufactured products	1.971441
21490020	Silver and platinum rolled, drawn or extruded semi-finished products	Other manufactured products	1.179909
21490030	Semi-manufactures of tungsten, molybdenum, tantalum, magnesium, cobalt, cadmium, titanium, zirconium and thallium	Other manufactured products	0.2978005
21490040	Non-ferrous (excl aluminium) metal powders and flakes	Other manufactured products	316.0247
21491700	Basic non-ferrous metal products - commission production (2141-2149)	Other manufactured products	1.044385
21491970	Wastes and scraps from the manufacture of non-ferrous metal products (incl precious) (2141-2149)	Other manufactured products	2.828047
22100010	Iron or steel pieces roughly shaped by forging	Other manufactured products	1.169981

IOPC (1284)	Sector name	Aggregated Sector	Intensity (kg/$)
22100020	Forged iron or steel tyres and wheels for railway or tramway locomotives and rolling stock	Other manufactured products	1.262435
22100030	Iron or steel chain (other than articulated link chain) and other forged articles of iron or steel	Other manufactured products	1.379882
22101700	Forged iron or steel products - commission production	Other manufactured products	1.199117
22101900	Repairing and servicing (2210)	Services	122.8897
22210010	Fabricated & prefabricated construction steel (incl scaffolding, perforated plate & ready made parts for structures)	Other manufactured products	0.9003789
22210020	Reinforcing steel rods or bars	Other manufactured products	0.8623627
22210030	Reinforcing welded steel mesh	Other manufactured products	0.8706325
22220010	Prefabricated metal or metal framed buildings (excl aluminium) and other transportable buildings	Other manufactured products	0.9226877
22220020	Aluminium or aluminium framed prefabricated buildings	Other manufactured products	0.9456678
22230010	Aluminium/aluminium framed doors (incl roller/concertina) & windows (incl glass); door/window frames; roller grilles	Other manufactured products	0.8806904
22230020	Aluminium fire doors	Other manufactured products	0.9049054
22230030	Aluminium combined door-window units	Other manufactured products	0.8635075
22230040	Architectural aluminium products (excl sheet metal), for building nec	Other manufactured products	0.8942542
22230050	Aluminium roofing and guttering	Other manufactured products	0.9048589
22230060	Other articles of aluminium (excl ladders) nec	Other manufactured products	0.8854077
22240010	Metal roofing and guttering (excl aluminium)	Other manufactured products	0.8850051
22290010	Iron or steel window-frames; metal (excl aluminium) door or door frames	Other manufactured products	0.8794671
22290020	Wooden fire doors	Other manufactured products	0.8872719
22290030	Iron or steel fire doors; fabricated iron or steel stairs, balustrades and other architectural products (excl Aluminium)	Other manufactured products	0.875846
22291700	Structural metal products - commission production (2221-2229)	Other manufactured products	0.8660254
22291970	Waste from the manufacture of ferrous metal products nec	Other manufactured products	4.482549

IOPC (1284)	Sector name	Aggregated Sector	Intensity (kg/$)
22310010	Metal cylinders (incl aerosol containers) for compressed or liquified gas	Other manufactured products	0.8679254
22310020	Sheet metal reservoirs, vats, tanks and similar containers of a capacity exceeding 300 litres	Other manufactured products	0.8849453
22310030	Sheet metal vats and tanks of a capacity not exceeding 300 litres	Other manufactured products	0.8852663
22310040	Super heated water boilers & steam generators (incl parts) (excl central heating); condensers for vapour power units	Non-medical equipment and machinery	0.8878695
22310050	Non-electric hot water or low pressure steam central heating boilers	Non-medical equipment and machinery	0.8430742
22310060	Iron, steel or aluminium vats, tanks, capacity exc. 300 litres and containers for compressed or liquefied gas	Other manufactured products	0.9260343
22310070	Plate iron, steel and aluminium vats and tanks, capacity not exc. 300 litres (excl with mechanical or thermal equipment)	Other manufactured products	0.881493
22310090	Metal freight containers (excl stock crates)	Other manufactured products	0.7450897
22390010	Metal containers nec	Other manufactured products	0.8835776
22390020	Sheet metal milk and cream cans of a capacity not exceeding 300 litres	Other manufactured products	0.8849811
22390030	Sheet metal household containers (excl sanitary ware)	Other manufactured products	0.8943025
22390040	Metal vacuum flasks	Consumable supplies	0.9561888
22400010	Sheet metal ducting	Other manufactured products	0.8585619
22400020	Sheet metal sanitary ware	Consumable supplies	7.43671E-05
22400030	Sheet metal stoppers, caps, lids, capsules for bottles, threaded bungs, bung covers, seals & packing accessories nec	Other manufactured products	0.9066331
22400040	Sheet metal non-electric tableware, kitchenware or other household articles and parts (excl containers and sanitary ware)	Consumable supplies	0.9207821
22400050	Sheet metal machine guards (not designed for use with a particular machine)	Other manufactured products	0.8344911
22400060	Sheet metal products nec	Other manufactured products	0.8820016
22401700	Metal container and sheet metal products - commission production (2231-2240)	Other manufactured products	0.8782688

IOPC (1284)	Sector name	Aggregated Sector	Intensity (kg/$)
22910010	Iron or steel fencing wire (excl stranded or barbed)	Other manufactured products	0.5251066
22910020	Wire stranded, cables, cordage, ropes, plaited bands and slings (excl electrically insulated slings)	Other manufactured products	0.6633474
22910030	Springs (incl leaves for springs)	Other manufactured products	0.5930376
22910040	Nails, tacks, staples, spiked cramps, studs, spikes & pins (incl drawing & cotter pins) (excl metallic dowel pins)	Other manufactured products	0.5968484
22910050	Woven or linked wire fabric (excl mattress supports)	Other manufactured products	0.000104415
22910060	Welded wire fabric (excl reinforcing)	Other manufactured products	0.5705403
22910070	Iron or steel wire gates (cross-sectional dimension of wire 16mm or less)	Other manufactured products	0.2643486
22910080	Iron or steel articulated link chain and parts	Other manufactured products	0.5834533
22910090	Domestic metal wire products; copper cloth, grill, netting and fencing; barbed wire; other wire products	Other manufactured products	0.5814012
22920010	Metal nuts, bolts (incl expansion), screws, rivets, washers, dowel pins, masonry anchors and turnbuckles	Other manufactured products	0.6192496
22930010	Metal coating and finishing	Other manufactured products	0.5643911
22990010	Metal hand tools (incl gardening; excl power operated or pneumatic)	Other manufactured products	0.6464128
22990030	Cutlery, kitchen ware and table ware (excl solid silver or gold) nec	Consumable supplies	0.2018915
22990050	Knives and cutting blades for metal or wood working tools and machines	Other manufactured products	0.5771964
22990060	Metal hand tool accessories & attachments (incl screwdriver & drill bits)(excl twist drills, taps, dies, chasers)	Other manufactured products	0.6264833
22990070	Non-ferrous metal steam, gas and water fittings other than domestic (incl taps, cocks and valves)	Other manufactured products	0.5881142
22990080	Tube or pipe fittings (excl valves) (eg couplings, elbows, sleeves), of copper or nickel (incl alloys) or aluminium	Other manufactured products	0.5954373
22990090	Munitions and ammunition (incl cartridges)	Other manufactured products	0.6285197
22990100	Aluminium venetian blinds (incl plastic coated)	Other manufactured products	0.5784571

IOPC (1284)	Sector name	Aggregated Sector	Intensity (kg/$)
22990110	Metal blinds and awnings (excl aluminium venetian blinds)	Other manufactured products	0.5654458
22990120	Locks (incl parts/padlocks); keys; metal fittings for windows or doors (incl hinges and hydraulic door closures)	Other manufactured products	0.5881252
22990140	Firearms (incl parts)	Other manufactured products	0.8073509
22990150	Fire extinguishers	Other manufactured products	0.578074
22990160	Articles of tungsten, molybdenum, tantalum, magnesium, cobalt, cadmium, titanium, zirconium and thallium (incl wrought)	Other manufactured products	0.5977308
22990170	Non-electric lamps and lighting fittings (incl pressure and gas lanterns)	Other manufactured products	0.6161267
22990180	Woven wire, link mesh or wire spring mattress supports (excl upholstered)	Other manufactured products	0.008365488
22990190	Cigarette and other lighters	Other manufactured products	0.02989696
22990200	Fabricated metal products (incl ladders) nec	Other manufactured products	0.6184664
22991700	Other fabricated metal products - commission production (2291-2299)	Other manufactured products	0.5665414
22991900	Repairing and servicing (2291-2299)	Services	0.5731241
23110010	Finished motor vehicles with less than 10 persons capacity	Non-medical equipment and machinery	0.1053226
23110020	Finished motor vehicles with 10 or more person capacity	Non-medical equipment and machinery	0.1000435
23110030	Finished trucks, truck type vehicles, utilities and panel vans	Non-medical equipment and machinery	0.09741458
23110040	Unassembled motor vehicles nec	Other manufactured products	0.1834242
23110050	Chassis with engines for motor vehicles	Other manufactured products	0.1583874
23110060	Engines nec, for motor vehicles or tractors	Other manufactured products	0.1039215
23119000	Second hand motor vehicles	Non-medical equipment and machinery	0.4067949
23120010	Motor vehicle and truck bodies (coachwork)	Other manufactured products	0.1184738
23120020	Caravans, camper trailers and similar vehicles	Non-medical equipment and machinery	0.1152584
23120030	Agricultural self loading and unloading semi-trailers (incl tippers)	Non-medical equipment and machinery	0.09669254
23120040	Other semi-trailers for the transport of goods & materials (incl tankers, vans, transporters, stock crates & jinkers)	Non-medical equipment and machinery	0.09149634

IOPC (1284)	Sector name	Aggregated Sector	Intensity (kg/$)
23120050	Trailers for the transport of goods and materials (incl box trailers, boat trailers and horse floats)	Other manufactured products	0.09858159
23120060	Other trailers & semi-trailers nec (excl for the transport of goods & materials, & domestic type camper trailers)	Other manufactured products	0.09557684
23120070	Body panels for trucks and buses	Other manufactured products	0.1043867
23120080	Parts nec, for motor vehicle trailers and semi-trailers	Other manufactured products	0.1028839
23130010	Vehicle electric motors of an output not exceeding 37.5W; other DC motors and DC generators	Other manufactured products	0.0992105
23130020	Motor vehicle and truck air conditioners	Other manufactured products	0.07865344
23130030	Motor vehicle apparatus for making, breaking, protecting & making connections to/in electrical circuits (excl wiring)	Other manufactured products	0.1193925
23130040	Motor vehicle or motor cycle wiring harnesses	Other manufactured products	0.09033338
23130050	Motor vehicle, tractor or motor cycle starting, heaters, demisters, windscreen wipers; lighting/signalling equipment	Other manufactured products	0.1078382
23130060	Motor vehicle, tractor and motor cycle filament lamps and sealed beam lamps	Other manufactured products	0.06669434
23130070	Motor vehicle & tractor gauges, revolution & production counters, speed indicators, thermostats & similar instruments	Other manufactured products	0.09816877
23130080	Automotive insulated cable, wire or strip	Other manufactured products	0.09091364
23190010	Motor vehicle transmission assemblies	Other manufactured products	0.1364583
23190020	Cylinder blocks, pistons, connecting rods, valves	Other manufactured products	0.1280806
23190030	Fuel, lubricating or cooling medium pumps	Other manufactured products	0.1237155
23190040	Cranks, crank & cam shafts, gears and flywheels	Other manufactured products	0.1204735
23190050	Motor vehicle, tractor and truck gaskets	Other manufactured products	0.1092251
23190060	Motor vehicle body panels	Other manufactured products	0.1335149
23190070	Motor vehicle and tractor parts and equipment nec	Other manufactured products	0.1017442
23910010	Vessels of 50 tonnes gross and over (incl floating structures)	Non-medical equipment and machinery	0.4023342
23920010	Small boats (incl rowing or sail), yachts and canoes under 5 tonnes displacement (incl inflatable vessels and canoes)	Non-medical equipment and machinery	0.7671625

IOPC (1284)	Sector name	Aggregated Sector	Intensity (kg/$)
23920020	Boats and other vessels for pleasure or sport (over 5 but under 50 tonnes)	Non-medical equipment and machinery	0.9099886
23920030	Cruise ships, ferry and excursion boats, and other vessels under 50 tonnes nec for the transport of persons and goods	Non-medical equipment and machinery	0.5847414
23921700	Ships and boats - commission production (2391-2392)	Non-medical equipment and machinery	0.4205612
23921900	Repairing and servicing (2391-2392)	Services	0.4601688
23930010	Locomotives and trams (incl underframes); railway rolling stock	Non-medical equipment and machinery	0.4445272
23931700	Railway rolling stock - commission production	Non-medical equipment and machinery	131.7354
23931900	Repairing and servicing (2393)	Services	0.4484416
23940010	Aircraft and aircraft parts	Non-medical equipment and machinery	0.2037545
23941700	Aircraft - commission production	Non-medical equipment and machinery	0.2027851
23941900	Repairing and servicing (2394)	Services	0.2043646
23990010	Motorised tanks and other armoured fighting vehicles and parts	Non-medical equipment and machinery	0.1077021
23990020	Transport equipment, parts and accessories nec (incl motorcycles and motor scooters)	Non-medical equipment and machinery	0.126723
23990030	Repairing and servicing - (incl factory motor vehicle engine repair or replacements) (2311-2319, 2399)	Services	0.096677
23991700	Motor vehicles, other transport equipment and parts - commission production (2311-2319, 2399)	Non-medical equipment and machinery	124.6517
24110010	Cameras, image projectors and parts	Non-medical equipment and machinery	0.06944521
24110020	Photographic goods nec (excl sensitised photographic film, paper, plates & chemicals)	Other manufactured products	0.06011051
24110030	Objective lenses, filters and other mounted optical elements; microscopes (excl optical) and diffraction apparatus and parts nec	Medical and precision equipment	0.07111377
24110050	Ophthalmic instruments and appliances	Medical and precision equipment	0.07598836
24110060	Spectacle and contact lenses	Other manufactured products	0.05665691
24110070	Spectacles, sunglasses and frames	Other manufactured products	0.06675056

IOPC (1284)	Sector name	Aggregated Sector	Intensity (kg/$)
24120010	Surgical, medical equipment (excl X-ray) and appliances (incl artificial joints, limbs or eyes, pacemakers, mechanical dental chairs & needles or syringes)	Medical and precision equipment	0.06521459
24120020	X-ray medical equipment and parts or accessories	Medical and precision equipment	0.06317947
24120030	Hearing aids and parts or accessories	Medical and precision equipment	0.05861286
24190010	Gas or liquid meters (incl parts and accessories)	Other manufactured products	0.05644229
24190020	Parking meters, traffic signals and other signalling equipment	Non-medical equipment and machinery	0.07692804
24190030	Taxi meters	Other manufactured products	0.06463128
24190040	Watches (incl metal watch straps), watch cases, clocks and parts	Other manufactured products	0.06929742
24190050	X-ray equipment (excl medical) and parts or accessories	Non-medical equipment and machinery	0.1166146
24190060	Professional or scientific instruments, apparatus or models for demonstrational purposes only	Non-medical equipment and machinery	0.0901919
24190070	Surveying, physical or chemical analysis and other measuring, checking and testing instruments, appliances and parts	Medical and precision equipment	0.0889051
24190080	Radio and radar equipment, navigational aids, and radio remote control equipment	Non-medical equipment and machinery	0.07058817
24190090	Optical fibres, fibre bundles and cables (excl insulated)	Other manufactured products	0.05154342
24190100	Professional and scientific equipment nec	Medical and precision equipment	0.1552156
24191700	Professional and scientific equipment - commission production (2411-2419)	Medical and precision equipment	0.06213372
24191980	General government consumption of fixed capital (2411-2419)	Other	131.7308
24210010	Mainframe and super-computers	Non-medical equipment and machinery	0.05160159
24210020	Computer file servers and other multiple-user computer hardware	Non-medical equipment and machinery	0.06367088
24210030	Laptops, notebooks, personal digital assistants and other portable computers	Non-medical equipment and machinery	0.06438775
24210040	Desktop computers (PCs)	Non-medical equipment and machinery	0.06179139
24210050	Computer peripheral devices (incl monitors, keyboards, mice, joysticks, speakers, drives and burners)	Non-medical equipment and machinery	0.07048894

IOPC (1284)	Sector name	Aggregated Sector	Intensity (kg/$)
24210060	Printers and plotters	Non-medical equipment and machinery	0.05967873
24210070	Other computer hardware, computer peripherals and accessories nec	Non-medical equipment and machinery	0.06171999
24210080	Photocopying machines and parts	Non-medical equipment and machinery	0.05314334
24210090	Electronic machines with a calculating device (incl cash registers, postage-franking & ticket machines) & parts	Non-medical equipment and machinery	0.04564788
24210100	Typewriters, word processors, addressing machines, EFTPOS machines, coin counting machines and other office machinery	Non-medical equipment and machinery	0.03086286
24210110	Money-changing, cigarette, food, beverage and other automatic goods vending machines (excl refrigerated vending machines)	Non-medical equipment and machinery	0.06425321
24210120	Office machines, parts and accessories nec	Non-medical equipment and machinery	0.05598639
24220010	Line telephone and telegraph equipment (excl headphones and parts)	Non-medical equipment and machinery	0.06899668
24220020	Radio and television studio equipment (incl cameras), transmitters, radio transceivers, mobile, cellular & car phones (excl parts)	Non-medical equipment and machinery	0.06261068
24220030	Satellite receivers (excl parts)	Non-medical equipment and machinery	0.06128865
24220040	Remote monitoring alarm systems	Non-medical equipment and machinery	0.06185632
24220050	Intercom equipment	Non-medical equipment and machinery	0.04754015
24220060	Parts for radio and television studio equipment, transmitters and radio transceivers	Other manufactured products	0.06812075
24220070	Television antennae parts	Other manufactured products	0.05638372
24220080	Telecommunication equipment parts (incl parts for mobile phones and satellite receivers)	Other manufactured products	0.06160302
24290010	Television receiving sets (excl parts)	Non-medical equipment and machinery	0.06193638
24290020	Radio receiving sets (incl car radios and clock radios) (excl parts)	Non-medical equipment and machinery	0.04935142
24290030	Sound and video recording and reproducing equipment (incl CD players, record players) (excl parts)	Non-medical equipment and machinery	0.06116714
24290040	Loudspeakers and audio-frequency electric amplifiers (excl hearing aids and parts)	Non-medical equipment and machinery	0.06211994

IOPC (1284)	Sector name	Aggregated Sector	Intensity (kg/$)
24290050	Electric or electronic alarm systems (excl remote monitoring alarm systems) and parts	Non-medical equipment and machinery	0.1604467
24290060	Video games, poker machines and other coin or disc operated games	Non-medical equipment and machinery	0.06961936
24290070	Prepared unrecorded media for sound or video recording (incl blank CDs, magnetic tapes)	Non-medical equipment and machinery	0.05240691
24290080	Other audio and video equipment and accessories (excl parts)	Non-medical equipment and machinery	0.06091875
24290090	Parts for television and radio receiving sets, sound and video recording and reproducing equipment, other audio and video equipment	Other manufactured products	0.05708133
24290100	Electronic equipment and parts nec	Non-medical equipment and machinery	0.06391697
24291700	Computer and electronic equipment - commission production (2421-2429)	Non-medical equipment and machinery	0.04720252
24291980	General government consumption of fixed capital (2421-2429)	Other	131.7308
24310010	Uninsulated copper and aluminium stranded wire, ropes, cables, plaited bands and slings	Other manufactured products	0.186599
24310020	Co-axial cable and other co-axial electric conductors	Other manufactured products	0.1744586
24310040	Cable (excl co-axial or insulated optical fibre), wire and strip	Other manufactured products	0.1769488
24310050	Insulated optical fibre cable	Other manufactured products	0.1712085
24320010	Electric light or lamp bulbs or tubes (incl filament or fluorescent) (excl automotive)	Other manufactured products	0.1499124
24320020	Incandescent light fittings	Other manufactured products	0.1780888
24320030	Cold, discharge, arc, ultra violet, infra-red and other electric lights, torches and fittings nec	Other manufactured products	0.1723175
24320050	Illuminated signs, name-plates and sign-plates having a permanently fixed light source	Other manufactured products	0.1691685
24390010	Dry cell batteries	Other manufactured products	0.1860756
24390020	Automotive wet cell batteries	Other manufactured products	0.172131
24390030	Batteries nec and battery components	Other manufactured products	0.1743537
24390050	Transformers	Other manufactured products	0.1705954
24390060	Electric motors, generators, electric generating sets and rotary converters (incl parts) (excl automotive)	Other manufactured products	0.1205468

IOPC (1284)	Sector name	Aggregated Sector	Intensity (kg/$)
24390070	Electrical welding (incl arc) base metal wire, rods, tubes, plates and electrodes	Other manufactured products	0.1778013
24390090	Relays and relay sets for radio, telephones and telegraphic equipment	Other manufactured products	0.2031956
24390100	Electrical apparatus to switch, protect/connect circuits (incl boards & cabinets equipped with such)(excl inductors)	Other manufactured products	0.1930427
24390120	Inductors (incl chokes, ballasts used with lighting apparatus and current limiting regulators)	Other manufactured products	0.2158637
24390130	Electric soldering and welding (incl arc) irons, guns and other machines, apparatus and parts	Non-medical equipment and machinery	0.173208
24390140	Industrial or laboratory electric furnaces and ovens	Non-medical equipment and machinery	0.1957515
24390150	Electric heating resistors (excl carbon)	Non-medical equipment and machinery	0.173761
24390160	Electrical insulators nec and other electrical equipment and parts nec	Non-medical equipment and machinery	0.1752732
24391700	Electrical equipment - commission production (2431-2439)	Non-medical equipment and machinery	0.1700037
24410010	Solid fuel or gas portable barbecues	Non-medical equipment and machinery	0.1584485
24410020	Domestic stoves, ovens and rangehoods (incl gas, electric, solid fuel, oil or spirit fired)	Non-medical equipment and machinery	0.1321693
24410030	Domestic refrigerators and freezers	Non-medical equipment and machinery	0.1314688
24410040	Compressors for domestic refrigeration equipment	Other manufactured products	0.1300132
24410050	Clothes washing machines, drying cabinets, tumble driers and dishwashing machines	Non-medical equipment and machinery	0.1306328
24410060	Domestic food waste disposal units	Non-medical equipment and machinery	0.1317276
24490010	Domestic gas, electric, solid fuel, oil, spirit fired space heaters and non-electric warm air furnaces	Non-medical equipment and machinery	0.1302567
24490020	Domestic soil heating apparatus	Non-medical equipment and machinery	0.1337248
24490030	Domestic solar hot water collectors, systems and parts (incl systems with conventional backup sources)	Non-medical equipment and machinery	0.1287922
24490040	Domestic gas and other non-electric water heaters and hot water systems (excl solar) and parts	Non-medical equipment and machinery	0.1328532

IOPC (1284)	Sector name	Aggregated Sector	Intensity (kg/$)
24490050	Domestic electric water heaters or hot water systems and parts	Non-medical equipment and machinery	0.1346934
24490060	Domestic room air conditioners and coolers (excl fans)	Non-medical equipment and machinery	0.1362087
24490070	Compressors for domestic air conditioning equipment	Other manufactured products	0.126593
24490080	Domestic fans (incl table, floor, wall, window, ceiling or roof)	Non-medical equipment and machinery	0.1277512
24490090	Electro-thermic appliances nec	Non-medical equipment and machinery	0.1193784
24490100	Sewing machines, vacuum cleaners, food mixers and other domestic appliances and parts (nec)	Non-medical equipment and machinery	0.1355971
24491700	Domestic appliances - commission production (2441-2449)	Non-medical equipment and machinery	0.3692241
24510010	Pumps and pumping machinery (incl petrol bowsers and air or gas compressors)	Non-medical equipment and machinery	0.1608374
24510020	Pump and compressor parts nec	Other manufactured products	0.1769341
24520010	Complete air conditioning units nec (incl ducting etc); air conditioning compressors or parts (commercial or industrial)	Non-medical equipment and machinery	0.1536041
24520030	Refrigeration cabinets, coolrooms, beverage dispensing equipment (cooling), refrigerated vending machines and refrigeration equipment nec (commercial or industrial) (incl water coolers)	Non-medical equipment and machinery	0.1586361
24520050	Water heaters (commercial or industrial) and parts	Non-medical equipment and machinery	0.1662548
24520060	Space heating equipment (commercial or industrial) (incl parts) nec	Non-medical equipment and machinery	0.158658
24610010	Lawn mowers	Non-medical equipment and machinery	0.16064
24610020	Ploughing, seeding and planting equipment and parts (excl hand tools)	Non-medical equipment and machinery	0.1536115
24610030	Harvesting, threshing and haymaking machinery (incl straw or fodder balers or agricultural mowers)	Non-medical equipment and machinery	0.1528973
24610040	Agricultural wheeled tractors (excl crawler)	Non-medical equipment and machinery	0.1503684
24610050	Mechanical appliances and parts for projecting, dispersing or spraying liquids or powders (excl industrial spray guns and steam blasting)	Non-medical equipment and machinery	0.05899616

IOPC (1284)	Sector name	Aggregated Sector	Intensity (kg/$)
24610070	Dairy machinery	Non-medical equipment and machinery	0.2114666
24610080	Agricultural and horticultural machinery and parts nec	Non-medical equipment and machinery	0.1551398
24620010	Construction and earthmoving wheeled tractors	Non-medical equipment and machinery	0.1519423
24620020	Front end shovel loaders; mechanical shovels, excavators & shovel loaders with a 360 degree revolving superstructure	Non-medical equipment and machinery	0.1625041
24620030	Bulldozers & other moving, grading, scraping, excavating, compacting or extracting construction machinery nec	Non-medical equipment and machinery	0.161431
24620040	Buckets, shovels, grabs, grips, blades, bodies and cabs for construction vehicles and other construction and earthmoving machinery parts	Non-medical equipment and machinery	0.169422
24620060	Machinery for crushing, grinding, mixing or kneading earth, stones, ores or other mineral substances in solid form	Non-medical equipment and machinery	0.1651847
24620070	Mineral substances sorting, screening, separating, washing, mixing or kneading machinery and parts	Non-medical equipment and machinery	0.1590282
24620080	Mining or drilling machinery and parts (incl coal or rock cutters, boring, sinking or tunnelling machinery)	Non-medical equipment and machinery	0.1650369
24630010	Gas welding and cutting equipment (excl filler welding rods)	Non-medical equipment and machinery	0.1727985
24630020	Hand tools, power operated (incl portable electric hand tools) nec	Non-medical equipment and machinery	0.1534335
24630030	Machine-tools for working materials other than metal, wood; laser machine tools; hand tools with self-contained motor	Non-medical equipment and machinery	0.1669385
24630040	Converters, ingot moulds and ladles, casting machines, metal-rolling mills and rolls and parts	Other manufactured products	0.1840805
24630050	Machining centres & other wood & metal working machinery & parts nec (excl saw blades, metal moulds & dies)	Non-medical equipment and machinery	0.1635853
24630060	Saw blades	Other manufactured products	0.173886
24630070	Metal dies, die sets and moulds	Other manufactured products	0.1674344

IOPC (1284)	Sector name	Aggregated Sector	Intensity (kg/$)
24630080	Interchangeable tools for power-operated hand tools; sintered metal plates; rock drilling and earth boring tools	Non-medical equipment and machinery	0.1673854
24630090	Parts for hand tools with self-contained non-electric motors	Other manufactured products	0.1691906
24630100	Metal work and tool holders, heads for machine tools, and other machine tool accessories and parts	Other manufactured products	0.1613879
24690010	Non-domestic cooking or heating machinery for food or drinks	Non-medical equipment and machinery	0.141596
24690020	Cream separators; bakery machinery (excl ovens) and other food and beverage processing machinery and parts	Non-medical equipment and machinery	0.1446283
24690030	Distilling/rectifying plant; heat exchange units; centrifuges nec; gas liquefying or beverages filtering machinery	Non-medical equipment and machinery	0.1659932
24690040	Machinery for can and bottle washing, packing, wrapping, canning, bottling and sealing of food and drink	Non-medical equipment and machinery	0.1691096
24690050	Parts of machinery nec for cleaning food or drink cans or bottles, (incl parts of dishwashing machines nec), or for filling, closing, sealing, capsuling or labelling containers for food or drink	Other manufactured products	0.1804052
24690060	Other food and beverage processing machinery and parts nec	Non-medical equipment and machinery	0.1883689
24690070	Industrial machinery and parts for textile manufacture and treatment industries (excl industrial sewing machines)	Non-medical equipment and machinery	0.1621112
24690080	Office type sheet fed printing machinery, accessories and parts	Other manufactured products	0.2376534
24690090	Other textile, apparel and leather production machinery and parts nec (incl industrial sewing machines)	Non-medical equipment and machinery	0.1871885
24690100	Printing machinery and parts	Non-medical equipment and machinery	0.1715509
24690110	Tobacco machinery and parts; bakery and biscuit ovens; dryers for agricultural products	Non-medical equipment and machinery	0.1620364
24690120	Machinery and parts for paper or book-binding	Non-medical equipment and machinery	0.1662244

IOPC (1284)	Sector name	Aggregated Sector	Intensity (kg/$)
24690140	Special purpose machinery & parts nec	Non-medical equipment and machinery	0.1837808
24691700	Specialised machinery and equipment - commission production (2461-2469)	Non-medical equipment and machinery	0.1619135
24910010	Wheeled tractors (excl crawler, agricultural, construction and earthmoving)	Non-medical equipment and machinery	0.1582681
24910020	Powered store trucks (incl those used on railway station platforms and forklifts)	Non-medical equipment and machinery	0.1725466
24910030	Conveyors, continuous-action, for goods & materials	Other manufactured products	0.1565677
24910040	Hoists, cranes and other lifting, loading or unloading machinery	Non-medical equipment and machinery	0.1630107
24910050	Elevators, continuous-action, for goods & materials; escalators & moving walkways	Non-medical equipment and machinery	0.1710699
24910060	Materials handling equipment and parts nec	Non-medical equipment and machinery	0.1657365
24990010	Hydraulic and pneumatic motors and parts	Non-medical equipment and machinery	0.1796806
24990020	Rubber, plastics or hot glass working machines (incl parts)	Non-medical equipment and machinery	0.03158607
24990030	Non-electric industrial and laboratory furnaces, ovens (other than bakery or biscuit) and incinerators and parts	Non-medical equipment and machinery	0.03532859
24990040	Dishwashing machines other than household	Non-medical equipment and machinery	0.1627845
24990050	Engines nec, turbines and water wheels and parts	Non-medical equipment and machinery	0.1598137
24990060	Oil filters, petrol filters and air intake filters for internal combustion engines	Other manufactured products	0.158936
24990070	Bearings (incl parts)	Other manufactured products	0.1424349
24990080	Parts for centrifuges (incl centrifugal dryers); parts for liquid or gas filtering or purifying machinery	Other manufactured products	0.1568739
24990100	Other machinery, equipment and parts nec	Non-medical equipment and machinery	0.1715177
24991700	Other machinery and equipment - commission production (2451-2452, 2491-2499)	Non-medical equipment and machinery	0.1587471
25110010	Assembled domestic wooden chairs, upholstered seating with wooden or metal frames, wooden or predominantly wooden domestic furniture nec	Other furniture	0.3303903
25110020	Assembled non-domestic wooden or predominantly wooden seating and furniture nec	Other furniture	0.3336618

IOPC (1284)	Sector name	Aggregated Sector	Intensity (kg/$)
25110030	Unassembled or partly assembled domestic wooden chairs, upholstered seating with wooden or metal frames, wooden or predominantly wooden furniture nec	Other furniture	0.3486801
25110040	Unassembled or partly assembled non-domestic wooden chairs, furniture and parts nec	Other furniture	0.3317306
25110050	Wooden or predominantly wooden medical, dental, surgical or veterinary furniture	Medical and surgical furniture	0.4058052
25110060	Upholstered seats with frames of any material for transport equipment and parts of passenger transport seats	Other manufactured products	0.3269687
25120010	Cabinets (Audiovisual) - Metal framed	Other manufactured products	0.3423444
25120020	Assembled metal or predominantly metal domestic furniture (excl upholstered seating with metal frames)	Other furniture	0.1883887
25120040	Metal medical, dental (excl mechanical dental chairs), surgical or veterinary furniture	Medical and surgical furniture	0.3255231
25120080	Unassembled large scale sheet metal or fabricated metal storage structures and shelving	Other furniture	0.2260834
25120090	Unassembled or partly assembled domestic metal furniture and parts nec (excl upholstered seats with metal frames)	Other furniture	0.3344973
25120100	Unassembled or partly assembled non-domestic metal furniture and parts nec (excl upholstered seats with metal frames)	Other furniture	0.3273646
25120110	Metal furniture fittings	Other furniture	0.3214199
25120120	Metal seating for entertainment and sporting venues (incl assembled and unassembled)	Other furniture	0.3363222
25120130	Assembled metal non-domestic furniture nec (excl upholstered seating with metal frames)	Other furniture	0.3308435
25130010	Mattresses, of, or stuffed with rubber	Other furniture	0.3311575
25130030	Water mattresses	Other furniture	0.3279796
25130040	Mattresses (excl water-mattresses and those of or stuffed with rubber)	Other furniture	0.01169628
25130050	Mattress supports (excl unupholstered woven wire, link mesh, wire springs and those of or stuffed with rubber)	Other furniture	0.3334335

IOPC (1284)	Sector name	Aggregated Sector	Intensity (kg/$)
25190010	Other assembled domestic furniture nec (excl wooden, metal or plastic)	Other furniture	0.3384998
25190020	Other medical, dental, surgical or veterinary furniture (excl wooden, metal or plastic) and parts	Medical and surgical furniture	0.3341889
25190030	Other assembled non-domestic furniture nec (excl metal, wood and plastic)	Other furniture	0.3320486
25190040	Unassembled or partly assembled domestic furniture and parts nec (excl metal, wood and plastic)	Other furniture	0.008180954
25190050	Unassembled or partly assembled non-domestic furniture and parts nec (excl metal, wood and plastic)	Other furniture	0.3242081
25191700	Furniture - commission production (2511-2513, 2519)	Medical and surgical furniture	0.422034
25910010	Badges, coins and medals, sheet metal	Other manufactured products	0.7222973
25910020	Jewellery and silverware	Other manufactured products	0.2761623
25910030	Imitation jewellery (excl incorporating precious metal except as plating or as minor constituents)(excl watch straps)	Other manufactured products	0.2650662
25920010	Toys (excl fur or leather)	Other manufactured products	0.3147002
25920020	Other articles for funfair or table games (incl billiards, snooker or pool, pintables articles and accessories)	Other manufactured products	0.2065911
25920030	Sporting equipment and accessories (incl fishing gear and gloves specially designed for use in sport)	Other manufactured products	0.3507124
25990010	Paint brushes or rollers, accessories and parts	Other manufactured products	0.2215489
25990020	Hair brushes, nail brushes, toothbrushes (excl electric) and other brushes for personal use	Consumable supplies	0.2906924
25990030	Advertising signs, name-plates and sign-plates (excl electric and metal)	Other manufactured products	0.2880547
25990040	Pens, pencils and refills, crayons and chalk	Consumable supplies	0.3104089
25990050	Typewriter ribbons and ink pads	Consumable supplies	0.01611857
25990060	Umbrellas	Other manufactured products	0.2633205
25990070	Musical instruments (incl parts and accessories)	Other manufactured products	0.3056601
25990090	Metal (other than precious) statuettes and other ornaments	Other manufactured products	0.2410537

IOPC (1284)	Sector name	Aggregated Sector	Intensity (kg/$)
25990100	Articles of precious metal (incl articles for technical or laboratory use)(excl jewellery)	Medical and precision equipment	0.4524306
25990120	Manufacturing nec (incl non-textile based sutures)	Consumable supplies	0.2138149
25991700	Manufacturing products nec - commission production	Other manufactured products	0.1592134
26110010	Electricity generated from fossil fuels	Utilities	11.10444
26120010	Hydro-electricity	Utilities	11.71185
26190010	Electricity generation nec	Utilities	11.86576
26400010	Other electricity service income	Utilities	0.279855
26401500	Margin - Electricity transmission, distribution and on selling (2620-2640)	Utilities	0.2848615
27001500	Margin - gas distribution	Utilities	1.176633
28100010	Water, sewerage and drainage services	Utilities	0.4635338
28101980	General government consumption of fixed capital (2811, 2812)	Other	0.4493131
29000010	Waste collection (incl skip and portable toilet hire), treatment disposal remediation and materials recovery services	Utilities	0.7876772
29221980	General government consumption of fixed capital (2911-2922)	Other	0.7734796
30100010	Residential building construction	Services	0.2865009
30109010	Second hand residential buildings	Services	0.3471727
30200010	Non-Residential building construction	Services	0.269697
30201980	General government consumption of fixed capital (3020)	Other	0.2701075
30209010	Second hand non-residential buildings	Services	0.3471989
31010010	Road and bridge construction (excl repair and maintenance)	Services	0.2725652
31010020	Repair and maintenance - road and bridge	Services	0.2499872
31019010	Second hand roads and bridges	Services	0.3407955
31090010	Non-building construction nec	Services	0.2670519
31090020	Repair and maintenance - non-building construction nec	Services	0.2475901
31091980	General government consumption of fixed capital (3101-3109)	Other	131.7382
31099010	Second hand non-building construction nec	Services	0.3580346
32000010	Trade services repair and maintenance	Services	0.299288

IOPC (1284)	Sector name	Aggregated Sector	Intensity (kg/$)
32000020	Other construction trade services	Services	0.3013509
32001980	General government consumption of fixed capital (3211-3299)	Other	131.7383
37000010	Non-margin - wholesaling services (excl repairing and servicing)	Services	0.2349939
37001400	Margin on reexports - wholesaling services	Services	0.6438205
37001500	Margin - wholesaling services	Services	0.2778318
38000020	Auction room operations; Electronic procurement brokering services	Services	0.3088349
38001800	Wholesale commission on sales	Services	0.3228978
38001980	General government consumption of fixed capital (3311-3800)	Other	131.7353
43000010	Non-Margin - retailing services (excl repairing and servicing)	Services	0.2604962
43001500	Margin - retailing services	Services	0.2890396
43001800	Retail commission on sales	Services	0.26642
43001980	General government consumption of fixed capital (3911-4320)	Other	131.7354
44000010	Accommodation services	Services	0.7757772
45000010	Meal preparation and presentation	Services	0.8051789
45000020	Beverage serving service	Services	0.7180186
45000030	Takeaway food	Services	0.8937478
45000040	Catering services	Services	0.7597278
45000050	Net losses from gambling - Clubs, pubs, taverns and bars (Hospitality)	Services	0.7031039
45001500	Margin - food and beverage services (4511-4530)	Services	0.702557
46100020	Non-margin - road freight transport services (incl rental or hire of trucks with driver)	Services	0.4946476
46100030	Road vehicle towing services	Services	0.501755
46101600	Margin - road freight transport services	Services	0.6336855
46210010	Interurban or non-metropolitan bus transport services (incl long distance, charter and rural)	Services	0.6290609
46220010	Urban or metropolitan bus and tramway transport services (incl short distance, airport and school)	Services	0.5899691
46230010	Taxi transport services	Services	0.6331808
46230020	Rental or hire of passenger car with driver	Services	0.5473284

IOPC (1284)	Sector name	Aggregated Sector	Intensity (kg/$)
46230030	Rental or hire of passenger road vehicle nec (incl buses and coaches) with driver	Services	0.5489261
46230040	Urban road passenger transport services nec	Services	1.736808
46230050	Interurban road passenger transport services nec	Services	1.171245
47100020	Non-margin - railway freight transport services nec	Services	0.4549492
47101600	Margin - railway freight transport services	Services	2.574826
47200010	Urban railway (incl monorail) passenger transport services	Services	0.7111859
47200020	Interurban railway passenger transport services	Services	0.7566106
48100020	Non-margin - ocean and inland water freight transport services	Services	1.018922
48101600	Margin - ocean and inland water freight transport services	Services	1.027654
48200010	Local water transport services for passengers	Services	0.7524738
48200020	Long distance water transport services for passengers	Services	0.6482546
48200030	Rental or hire of water vessel with operator	Services	0.6329423
49000020	Non-margin - air and space freight transport services	Services	0.4108922
49000030	Air passenger transport services	Services	0.5451511
49000040	Rental or hire of aircraft with operator	Services	0.3834105
49001600	Margin - air and space freight transport services	Services	0.4232153
50100010	Scenic and sightseeing transport services	Services	0.9685037
50211600	Margin - pipeline transport services	Services	0.6066253
50290010	Transport services nec (incl ski lift operation)	Services	0.5443666
50291980	General government consumption of fixed capital (5010-5029)	Other	122.9449
51010010	Postal services	Services	1.70411
51020010	Courier pick-up and delivery services	Services	1.274308
51021980	General government consumption of fixed capital (5101-5102)	Other	122.9388
52100030	Non-margin - stevedoring and port handling services (5211-5219)	Services	0.2146658
52100040	Support services to water transport nec (5211-5219)	Services	0.2126557
52101400	Margin on reexports - services to water transport (5211-5219)	Services	0.2322024

IOPC (1284)	Sector name	Aggregated Sector	Intensity (kg/$)
52101600	Margin - services to water transport (5211-5219)	Services	0.282701
52200010	Airport operations and other air transport support services nec	Services	0.2093892
52910010	Customs agency services	Services	0.1829551
52920010	Freight forwarding agency services	Services	0.186133
52990010	Other transport support services nec	Services	0.185665
52990020	Support services for road transport nec (incl taxi radio base and road vehicle driving service)	Services	0.1967543
52990030	Support services for railway transport nec (incl station and terminal operations)	Services	0.1979814
52991980	General government consumption of fixed capital (5211-5299)	Other	0.1852453
53010010	Grain storage services	Services	0.188871
53090010	Warehousing and storage services nec	Services	0.1922589
53091980	General government consumption of fixed capital (5301-5309)	Other	0.1862813
54110010	Newspaper publishing (incl printed and published by the same business) published once a week or more	Services	0.1728359
54110020	Newspaper publishing (incl printed and published by the same business) published less than weekly	Services	0.1545854
54110030	Newspapers - advertising services	Services	0.1386433
54110040	Copyright leasing - newspapers	Services	0.1777725
54120010	Magazine and other periodical publishing (incl printed and published by the same business)	Services	0.2046676
54120030	Magazines and other periodicals - advertising services	Services	0.1410081
54120040	Copyright leasing - magazines and other periodicals	Services	0.1857111
54130010	Book publishing (incl textbooks, encyclopedias, travel guides and atlases) (incl printed and published by the same business)	Services	0.2133875
54130030	Books - advertising services	Services	0.1343865
54130040	Copyright leasing - books	Services	0.1835912
54140010	Directory, mailing list, collection or compilation publishing (incl printed and published by the same business)	Services	0.259078

IOPC (1284)	Sector name	Aggregated Sector	Intensity (kg/$)
54140030	Directory, mailing list, collection or compilation - advertising services	Services	0.1460118
54190010	Other publishing nec (incl maps, greeting cards, postcards and calendars) (incl printed and published by the same business)	Services	0.1913923
54190030	Other publishing - advertising services	Services	0.144792
54190040	Copyright leasing - other	Services	0.3196126
54200010	Software publishing services (non-customised)	Services	0.1643713
54200020	Copyright leasing - software (non-customised)	Services	0.1732045
54201980	General government consumption of fixed capital (5411-5420)	Other	0.1479464
55110010	Motion picture and video production	Services	0.1994239
55120010	Motion picture and video distribution services	Services	0.198962
55120020	Copyright leasing - motion pictures and videos	Services	0.1997035
55130010	Motion picture theatre services	Services	0.2317761
55140010	Post-production services and other motion picture and video activities	Services	0.2413199
55210010	Music publishing nec (incl sheet music)	Services	0.2304221
55210030	Music copyrights (Acquiring, registering and selling)	Services	0.2416744
55220010	Music and other sound recording studios operation (incl pre-recorded radio programming services)	Services	0.2527681
55221980	General government consumption of fixed capital (5511-5522)	Other	0.1994338
56100010	Radio broadcasting services	Services	0.1890683
56210010	Free-to-air television broadcasting services	Services	0.1740487
56220010	Cable (Pay TV) and other subscription broadcasting services	Services	0.180292
56221980	General government consumption of fixed capital (5610-5622)	Other	0.1720571
57000010	Internet publishing and broadcasting services (incl radio, television, books, newspapers and magazines)	Services	0.08646411
57000020	Internet publishing - advertising services	Services	0.08424482
57001980	General government consumption of fixed capital (5700)	Other	122.9502

IOPC (1284)	Sector name	Aggregated Sector	Intensity (kg/$)
58010020	Wired telecommunications network services (incl International, long distance and local)	Services	0.301601
58020010	Mobile and other telecommunication network services nec (incl wireless and satellite)	Services	0.3040778
58090010	Other telecommunications services nec	Services	0.3192903
58091980	General government consumption of fixed capital (5801-5809)	Other	122.997
59100010	Internet access (incl ISPs) and internet search services	Services	0.0920035
59210020	Data processing and web hosting services	Services	0.1001966
59220010	Information storage and retrieval services	Services	0.0989796
59221980	General government consumption of fixed capital (5910-5922)	Other	122.9502
60100010	Library and archive services	Services	0.06833852
60200010	Other information services (incl radio and television new collection and telephone based recorded information services)	Services	0.1285567
60201980	General government consumption of fixed capital (6010-6020)	Other	0.05885996
62000010	Bank services - Financial intermediation services indirectly measured	Services	0.05238305
62000020	Bank services nec	Services	0.05489969
62220020	Building society services nec	Services	0.05524855
62230020	Credit union services nec	Services	0.05192867
62290010	Other depository financial services (incl securitiser services) - Financial intermediation services indirectly measured	Services	0.05084292
62290030	Other depository financial services nec (incl money market and securitiser services)	Services	0.0504161
62300010	Non-depository finance services - Financial intermediation services indirectly measured	Services	0.05007433
62300020	Non-depository finance services nec	Services	0.0788534
62400010	Financial asset investors	Services	0.05041781
63100010	Life insurance provision	Services	0.1204979
63210010	Health insurance provision	Services	0.120459
63220010	Fire and industrial special risks insurance provision	Services	0.1246344

IOPC (1284)	Sector name	Aggregated Sector	Intensity (kg/$)
63220020	Houseowner and household insurance provision	Services	0.1206426
63220030	Motor vehicle comprehensive and compulsory third party insurance provision	Services	0.118255
63220040	Public liability, product liability and professional indemnity insurance provision	Services	0.1119603
63220060	Marine insurance provision (Non-margin); aviation hull/cargo insurance provision	Services	0.1678034
63220070	Employers liability insurance provision	Services	0.3207901
63220080	Insurance provision (incl travel insurance) nec.	Services	0.1148744
63221500	Marine insurance provision (Margin)	Services	0.3016036
63300010	Superannuation fund services	Services	0.1206748
63301980	General government consumption of fixed capital (6310-6330)	Other	122.9979
64110010	Financial asset broking services	Services	0.1156733
64190010	Insurance fund management service	Services	0.1090461
64190020	Auxiliary services to finance and investment nec	Services	0.1113381
64200010	Services to insurance nec	Services	0.1126736
66110010	Passenger car rental or hire (incl cars, minibuses) - (excl financial leases)	Services	0.5286387
66190010	Other motor vehicle rental or hire (incl caravan & trailer) (excl financial leases) nec	Services	0.3016652
66190020	Transport equipment rental or hire (incl ship & boat) nec	Services	0.3120051
66200010	Farm animal and bloodstock leasing	Services	131.3804
66310010	Heavy machinery and Scaffolding (excl erection) rental or hire (excl financial leases)	Services	0.2781562
66320010	Video and other electronic media rental or hire	Services	0.3084361
66390010	Other goods and equipment rental hire nec (incl art works, household goods and office machinery)	Services	0.2806153
66400010	Non-financial intangible assets (excl copyrights) leasing	Services	0.2916998
66401980	General government consumption of fixed capital (6611-6640)	Other	131.7409
67110010	Ownership of dwellings	Services	0.07456128
67110020	Residential caravan park operation and residential property body corporate or strata corporation services	Services	0.07343449

IOPC (1284)	Sector name	Aggregated Sector	Intensity (kg/$)
67111980	General government consumption of fixed capital (6711)	Other	0.1445212
67120010	Non-residential property operator services (incl non-residential property body corporate or strata corporation services)	Services	0.1681234
67200010	Real estate agent services	Services	0.1659544
67200020	Agricultural or pastoral property broking, leasing, renting or valuing	Services	0.1667639
67201980	General government consumption of fixed capital (6712-6720)	Other	131.7382
69000020	Architectural services	Services	0.1400199
69000030	Surveying and mapping services	Services	0.1411885
69000040	Quantity surveying services	Services	0.1407761
69000050	Engineering design and consulting services	Services	0.1420864
69000060	Commercial art and display services	Services	0.1406477
69000070	Specialised design services nec (incl fashion, interior and jewellery design)	Services	0.1475477
69000080	Scientific testing and analysis services	Services	0.1449104
69000090	Legal services	Services	0.1453405
69000100	Accounting services	Services	0.1488046
69000110	Advertising services	Services	0.1413023
69000120	Market research services	Services	0.1410389
69000130	Statistical services	Services	0.1401274
69000140	Corporate head office management	Services	0.1407902
69000150	Management services (incl business, artists, entertainers and sporting professionals)	Services	0.1833092
69000160	Management advice and consulting services nec (excl financial and computer consulting)	Services	0.1436987
69000170	Veterinary services	Services	0.1442516
69000180	Photography services nec (incl Video filming of Weddings etc)	Services	0.1435543
69000190	Meteorology services	Services	0.1443386
69000200	Interpreting and translating services	Services	0.1485771
69000210	Research and development services	Services	0.1533312
69000220	Own account research and development	Services	0.1409529
69000230	Professional, scientific or technical services nec	Services	0.1432064

IOPC (1284)	Sector name	Aggregated Sector	Intensity (kg/$)
69001980	General government consumption of fixed capital (6910, 6921-6950, 6961-6999)	Other	0.14042
70000010	Computer systems, hardware and software design and development services	Services	0.1086178
70000030	Computer support services	Services	0.1086462
70001980	General government consumption of fixed capital (7000)	Other	0.1034368
72110010	Employment placement and recruitment services (incl casting agency service)	Services	0.1041927
72120010	Labour supply services	Services	0.1060685
72200010	Travel agency and tour arrangement services	Services	0.1148193
72910010	Periodical subscription service	Services	0.1212103
72910020	Office administration services nec (incl clerical, billing, record-keeping and payroll services)	Services	0.1091538
72920010	Document preparation services nec (incl word processing, stenography, typing, transcription and resume writing)	Services	0.1310565
72930010	Credit rating, credit investigation and collection agency services	Services	0.1054026
72940010	Call centre operation	Services	0.1676536
72990010	Tourist information centre operation	Services	0.1074526
72990020	Theatre, concert and sport ticketing and booking services	Services	0.1095814
72990030	Event management or promotion (incl sport, art or similar); fund raising services (fee based) or administration services nec	Services	0.1252502
72991980	General government consumption of fixed capital (7211-7299)	Other	0.1072812
73110010	Building and other industrial cleaning services nec (incl gutters, drains, roads, beaches, swimming pools and toilets)	Services	0.1060595
73120010	Pest control services	Services	0.1090343
73130010	Gardening services	Services	0.1082895
73200010	Crating or packing services for transport	Services	0.1037596
73200020	Packaging of fresh produce, groceries; bottling or rebottling services and packaging services nec	Services	0.1071489

IOPC (1284)	Sector name	Aggregated Sector	Intensity (kg/$)
73201980	General government consumption of fixed capital (7311-7320)	Other	131.7354
75000010	Government administration and regulatory services	Other	0.3142759
75400010	Judicial services	Services	0.3135832
75510010	Domestic government diplomatic and consular services	Other	0.3179826
75521980	General government consumption of fixed capital (7510-7552)	Other	0.313333
76000010	Defence services	Services	0.1397085
76001980	General government consumption of fixed capital (7600)	Other	0.1394573
77110010	Police services	Services	0.1140756
77120010	Investigative and security services (incl locksmiths) (excl police)	Services	0.1126576
77130010	Fire brigade services (incl forest fire fighting)	Services	0.1110733
77140010	Correctional and detention centres (incl juvenile)	Services	0.1108484
77190010	Public order and safety services (incl coastwatch and country border) nec	Services	0.1145493
77201980	General government consumption of fixed capital (7711-7720)	Other	0.1110574
80100010	Preschool education services	Services	0.1061411
80200010	Primary education services	Services	0.1043588
80200020	Secondary education services	Services	0.1050793
80200030	Special school education services	Services	0.1038904
81010010	Technical, vocational and other non-tertiary education services	Services	0.1357763
81020010	Tertiary higher education services (incl undergraduate and postgraduate)	Services	0.1508269
82110010	Sports or physical recreation instruction services (incl ski or snowboard) nec	Services	0.102655
82120010	Arts education services (excl vocational)	Services	0.1013588
82190010	NPISH-provided adult, community and other education services	Services	0.3020907
82190020	Other adult, community and other education services	Services	0.12139
82200010	Educational support services	Services	0.1083446

IOPC (1284)	Sector name	Aggregated Sector	Intensity (kg/$)
82201980	General government consumption of fixed capital (8010-8220)	Other	0.1045349
84010010	Hospital services (except Psychiatric Hospitals)	Services	0.1262533
84020010	Psychiatric Hospitals services	Services	0.1262006
85110010	General practice medical services	Services	0.1258666
85120010	Specialist medical services	Services	0.1251147
85200010	Pathology and Diagnostic Imaging services	Services	0.1260949
85310010	Dental services	Services	0.1246277
85320010	Optometry and optical dispensing	Services	0.124658
85330010	Physiotherapy services	Services	0.124594
85340010	Chiropractic and Osteopathic Services	Services	0.1236748
85390010	NPISH-provided other allied health services	Services	0.515165
85390020	Other allied health services n.e.c.	Services	0.1251927
85910010	Ambulance services	Services	0.1257522
85990010	Other health services nec	Services	0.1232873
85991980	General government consumption of fixed capital (8401 - 8599)	Other	0.127529
86010010	Residential care services for the elderly (Aged care)	Services	0.1030823
86010020	Residential care services for the disabled	Services	0.1019821
86090010	Other residential care services (incl mental health illnesses or substance abuse) nec	Services	0.1019573
87100010	Child care services	Services	0.1034273
87900010	NPISH-provided other social assistance services n.e.c. (incl elderly, disabled, marriage and adoption services)	Services	0.5336632
87900020	Other social assistance services n.e.c. (incl elderly, disabled, marriage and adoption services)	Services	0.1033187
87901980	General government consumption of fixed capital (8601 - 8790)	Other	0.1040298
89100010	Museum and art gallery services	Services	0.1341729
89210010	Zoological and botanical services	Services	0.1341531
89220010	Nature reserve and conservation park services	Services	0.1305035
89221980	General government consumption of fixed capital (8910-8922)	Other	0.124885
90010010	Performing arts operation nec (incl theatre restaurants and circuses)	Services	0.134499

IOPC (1284)	Sector name	Aggregated Sector	Intensity (kg/$)
90020010	Services of independent creative artists, writers and performers	Services	0.1235888
90020020	Theatre lighting, costume design and set design services	Services	0.1223344
90030010	Performing arts venue operation	Services	0.1457943
90031980	General government consumption of fixed capital (9001-9003)	Other	0.1258909
91110010	Gymnasia or fitness centre operation	Services	0.1326587
91120010	Sports professional services	Services	0.1267641
91120020	NPISH-provided sport and physical recreation club operation	Services	0.3305276
91120030	Other sport and physical recreation club operation	Services	0.1315221
91130010	Sports grounds and similar recreational facilities operation nec (excl Gymnasia or fitness centre)	Services	0.1291157
91140010	NPISH-provided sport and physical recreation administrative services	Services	0.3386664
91140020	Other sport and physical recreation administrative services	Services	0.1295932
91210010	Horse and dog racing, administration and track operation	Services	0.1256471
91290010	racing horse or dog training and stable (kennel) operation	Services	0.1336525
91310010	Amusement parks and centres operation	Services	0.1366411
91390010	Amusement and other recreational activities nec	Services	0.1479572
91391980	General government consumption of fixed capital (9111-9139)	Other	0.128223
92010010	Casinos operation	Services	0.1288817
92020010	Lottery operation	Services	0.1333157
92090010	Totalisator agency services	Services	0.1281147
92090020	Gambling services nec	Services	0.1338869
92091980	General government consumption of fixed capital (9201-9209)	Other	132.7667
94110010	Automotive electrical repair or replacement services	Services	0.1458281
94110020	Automotive electrical installation services	Services	0.1470905
94120010	Car wash and cleaning services	Services	0.1481611
94120020	Automotive body, paint and interior repair services	Services	0.152863

IOPC (1284)	Sector name	Aggregated Sector	Intensity (kg/$)
94190010	Other automotive repair services	Services	0.1463319
94191980	General government consumption of fixed capital (9411-9419)	Other	131.7353
94210010	Domestic appliance repair and maintenance	Services	0.1265389
94220010	Electronic and precision equipment repair and maintenance (excl domestic appliance)	Services	0.1240848
94290010	Other machinery and equipment repair and maintenance	Services	0.1245155
94910010	Clothing and footwear repair	Services	0.1317865
94990010	Other repair and maintenance nec	Services	0.1258155
94991980	General government consumption of fixed capital (9421-9499)	Other	131.7353
95110010	Hairdressing and beauty services (incl massage services nec)	Services	0.2376698
95200010	Funeral directing services	Services	0.2334064
95200020	Crematoria and cemetery services	Services	0.2051987
95300010	Personal services nec (incl weight reduction centres and prostitution services	Services	0.2331309
95310010	Laundry and dry-cleaning services	Services	0.2191892
95320010	Photographic film processing	Services	0.2141073
95330010	Parking services	Services	0.2316813
95391980	General government consumption of fixed capital (9511-9539)	Other	0.2062064
95400010	Religious services	Services	0.09896035
95510010	Business and professional association services	Services	0.1052575
95520010	Labour association services	Services	0.1025439
95590010	Interest groups nec (incl welfare fundraising services)	Services	0.1035715
95590020	Services to students at post-secondary institutions by their sports and student unions	Services	0.1034714
95591980	General government consumption of fixed capital (9540-9559)	Other	131.322
96000010	Domestic services of private household employees	Services	0.2415544
